# The Hemagglutinin-Neuraminidase (HN) Head Domain and the Fusion (F) Protein Stalk Domain of the Parainfluenza Viruses Affect the Specificity of the HN-F Interaction

**DOI:** 10.3389/fmicb.2018.00391

**Published:** 2018-03-13

**Authors:** Masato Tsurudome, Junpei Ohtsuka, Morihiro Ito, Machiko Nishio, Tetsuya Nosaka

**Affiliations:** ^1^Department of Microbiology and Molecular Genetics, Graduate School of Medicine, Mie University, Tsu, Japan; ^2^Department of Biomedical Sciences, College of Life and Health Sciences, Chubu University, Kasugai, Japan; ^3^Department of Microbiology, Wakayama Medical University, Wakayama, Japan

**Keywords:** parainfluenza virus 2, human, parainfluenza virus 5, simian virus 41, hemagglutinin-neuraminidase protein, fusion protein, cell–cell fusion

## Abstract

Membrane fusion by the parainfluenza viruses is induced by virus-specific functional interaction between the attachment protein (HN) and the fusion (F) protein. This interaction is thought to be mediated by transient contacts between particular amino acids in the HN stalk domain and those in the F head domain. However, we recently reported that replacement of specified amino acids at or around the dimer interface of the HN head domain remarkably affected the F protein specificity. We then intended to further investigate this issue in the present study and revealed that the HPIV2 HN protein can be converted to an SV41 HN-like protein by substituting at least nine amino acids in the HPIV2 HN head domain with the SV41 HN counterparts in addition to the replacement of the stalk domain, indicating that specified amino acids in the HN head domain play very important roles in determining the specificity of the HN-F interaction. On the other hand, we previously reported that the PIV5 F protein can be converted to an SV41 F-like protein by replacing 21 amino acids in the head domain of the PIV5 F protein with those of the SV41 F protein. We then intended to further investigate this issue in the present study and found that replacement of 15 amino acids in the stalk domain in addition to the replacement of the 21 amino acids in the head domain of the PIV5 F protein resulted in creation of a more SV41 F-like protein, indicating that specified amino acids in the F stalk domain play important roles in determining the specificity of the HN-F interaction. These results suggest that the conformations of the HN stalk domain and the F head domain are dependent on the structures of the HN head domain and the F stalk domain, respectively. Presumably, the conformations of the former domains, which are considered directly involved in the HN-F interaction, can be modified by subtle changes in the structure of the latter domains, resulting in an altered specificity for the interacting partners.

## Introduction

The parainfluenza viruses are classified into three genera in the subfamily *Paramyxovirinae*, that is, *Rubulavirus, Avulavirus*, and *Respirovirus* (Karron and Collins, 2013; Lamb and Parks, 2013). The human parainfluenza virus 2 (HPIV2), HPIV4A, HPIV4B, simian virus 41 (SV41), and parainfluenza virus 5 (PIV5) are members of the genus *Rubulavirus*, while HPIV1 and HPIV3 belong to the genus *Respirovirus*. Newcastle disease virus (NDV) is a member of the avian paramyxoviruses of the genus *Avulavirus* (Lamb and Parks, 2013; International Committee on Taxonomy of Viruses [ICTV], 2016). Although mumps virus (MuV) belongs to the genus *Rubulavirus*, it is not regarded as a parainfluenza virus (Karron and Collins, 2013). The parainfluenza viruses have two kinds of glycoprotein spikes on the envelope: the hemagglutinin-neuraminidase (HN) and the fusion (F) protein (Karron and Collins, 2013). The homotrimeric F protein mediates membrane fusion such as cell–cell fusion or virus-cell fusion; cleavage of the F precursor (F_0_) by cellular proteases into disulfide-linked F_1_ and F_2_ subunits is necessary for its fusion activity (Karron and Collins, 2013; Lamb and Parks, 2013). On the other hand, the HN protein is a homotetramer which consists of two dimers and is responsible for binding to the sialoconjugate receptors on the cell surface and for enzymatic destruction of the receptors (Lamb and Parks, 2013). Importantly, the HN protein is required for the F protein in order to mediate membrane fusion, although it is not precisely known how the HN protein activates the F protein. It is appreciated, at least, that membrane fusion is induced through a series of conformational changes of the F protein that has been triggered by specific interaction with the cognate HN protein (Lamb and Jardetzky, 2007; Iorio et al., 2009).

The HN head domain carries both the receptor-binding and -destroying activities (Thompson and Portner, 1987; Mirza et al., 1993; Yuan et al., 2005). On the other hand, the HN stalk domain harbors the site that determines the F protein specificity in promoting cell–cell fusion and thus would be involved in the functional interaction with the F protein (Tsurudome et al., 1995; Tanabayashi and Compans, 1996; Deng et al., 1997); in the case of the PIV5 HN protein, a putative F-activating region (FAR) has been identified in the stalk domain (Bose et al., 2014). According to the model based on the structural studies on the PIV5 and NDV HN proteins (Bose et al., 2015), the HN protein tetramer converts from the “4-heads-down” conformation to the “4-heads-up” conformation after interacting with the receptors, by which the otherwise hidden FAR in the stalk domain is exposed and becomes accessible to the F protein.

Since the headless HN proteins of PIV5, SV41, NDV, and MuV have been found to efficiently activate their cognate F proteins and induce extensive cell–cell fusion (Bose et al., 2012, 2014; Tsurudome et al., 2015), the HN stalk domain seems to harbor sufficient elements for interacting with the F protein and activating it. However, we have recently found that the primary structures of the HN stalk domains cannot explain the unidirectional substitutability among the rubulavirus HN proteins (Tsurudome et al., 2015). We also found that replacement of specified amino acids at or around the dimer interfaces of the HN head domain remarkably modify the F protein specificity, suggesting that changes in the head domain structure somehow alter the conformation of the stalk domain, thereby converting the F protein specificity (Tsurudome et al., 2015).

As for the region of the F protein that would be responsible for the interaction with the HN stalk domain, Bose et al. and we have recently identified such regions at different locations in the PIV5 F head domain (Bose et al., 2013; Tsurudome et al., 2013). During this process, we created a chimeric PIV5 F protein by replacing 21 amino acids of the head domain with the SV41 F counterparts. This chimeric PIV5 F protein, no. 36, was efficiently activated by the SV41 HN protein and induced extensive cell–cell fusion, but not by the PIV5 HN protein. On the other hand, we previously obtained a chimeric HPIV2 HN protein, CH95-571, by replacing the cytoplasmic/transmembrane domains and the stalk domain with those of the SV41 HN protein; CH95-571 activated the SV41 F protein nearly 10 times as efficiently as it activated the HPIV2 F protein (Tsurudome et al., 1995). Surprisingly, it was shown that the SV41 HN-like protein, CH95-571, failed to activate the SV41 F-like protein, no. 36 (Tsurudome et al., 2015), suggesting that the SV41 HN-derived stalk domain of CH95-571 and/or the SV41 HN-derived 21 amino-acid region in the head domain of no. 36 do not have enough elements to mediate functional HN-F interaction between these “SV41-like” HN and F proteins. Indeed, by replacing some amino acids around the dimer interface of the HPIV2 HN-derived head domain of CH95-571 with those of the SV41 HN protein, we succeeded in creating several chimeric HN proteins that were able to activate both no. 36 and the SV41 F protein (Tsurudome et al., 2015). We further investigated this issue in the present study and eventually obtained various SV41 HN-like chimeric HPIV2 HN proteins and an SV41 F-like chimeric PIV5 F protein which induced cell–cell fusion upon co-expression.

## Materials and Methods

### Cells and Antibodies

BHK-21 (BHK) cells (ATCC: CCL-10) were maintained in Eagle’s minimum essential medium supplemented with 5% fetal calf serum. The monoclonal antibodies MAb 173-1A and MAb 127A-1, specific for the HPIV2 HN protein and the SV41 HN protein, respectively, were reported previously (Tsurudome et al., 1989, 1990). The epitopes for MAb 173-1A and MAb 127A-1 reside in the HPIV2 HN protein at residues from 355 to 360 and in the SV41 HN protein at residues from 326 to 335, respectively, as analyzed by indirect immunofluorescence assay using chimeric HN proteins of HPIV2 and SV41. The monoclonal antibody MAb 1D1 is specific for the PIV5 F protein whose epitope resides at residues from 458 to 452 (Tsurudome et al., 2006). The culture supernatant of hybridoma cells secreting each antibody was used without dilution. The rabbit antiserum specific for the PIV5 F_2_ subunit (Tsurudome et al., 2001) was used after diluting 1:16 with phosphate-buffered saline pH 7.4 (PBS). Anti-β-actin monoclonal antibody, which was used after diluting at 1:1000, and biotinylated horse immunoglobulin specific for mouse IgG were purchased from MBL (Medical and Biological Laboratories, Japan) and Vector Laboratories, respectively.

### Plasmid Vectors

The pcDL-SRα expression vectors encoding the HN and F proteins of HPIV2, SV41, PIV5, and the chimeric HN proteins (CH95-571, CH148-209, CH148-294, CH210-294, CH295-447, and CH5-41) were reported previously (Tsurudome et al., 1995, 2011). The pcDL-SRα expression vectors encoding the chimeric HN protein IM18 was reported elsewhere (Tsurudome et al., 2015). The pcDL-SRα expression vectors encoding the chimeric HN proteins CH95-209 and CH448-571 were created in the current study as described previously (Tsurudome et al., 1995).

### Creation of Chimeric HN and F Proteins

To create chimeric proteins of the SV41 HN protein and IM18, desired portions of the pcDL-SRα expression vectors encoding either of the proteins were amplified and connected by PCR. Then the connected PCR product was inserted into the IM18-encoding pcDL-SRα expression vector (between the restriction enzyme sites EcoR I and Pvu II) by using In-Fusion HD Cloning Kit (Clontech Laboratories). To create the chimeric HN protein CH295-415, desired portions of the pcDL-SRα expression vector encoding the HN protein of HPIV2 or SV41 were amplified, joined by PCR, and inserted into the SV41 HN-encoding pcDL-SRα expression vector (between the two Pvu II sites) by using the In-Fusion HD Cloning Kit. The chimeric protein no. 36 was reported previously (Tsurudome et al., 2013). To create chimeric proteins of the SV41 F protein and no. 36, desired portions of the pcDL-SRα expression vector encoding either of the proteins were amplified and connected by PCR. Then the connected PCR products was inserted into the no. 36-encoding pcDL-SRα expression vector by using the restriction enzyme sites Spe I, Bsp1407I, Pst I, and EcoR I. The nucleotide sequences of the primers for PCR and those for In-Fusion ligation are presented in **Supplementary Figures S1, S2**.

### Quantification of Cell–Cell Fusion

Subconfluent BHK cells (ca. 3.0 × 10^6^) in one well of six-well culture plate were transfected with 0.2 or 1.0 μg/well of the pcDL-SRα expression vector encoding each HN protein together with 2 μg/well of the pcDL-SRα expression vector encoding each F protein by using X-tremeGENE 9 DNA transfection kit (Roche Diagnostics). After 12 h or 24 h of incubation at 37°C, the cells were fixed with 4% paraformaldehyde in PBS, washed three times with PBS, treated with methanol, and stained with Giemsa’s solution (Sigma). A photomicrograph, which corresponded to an area of 0.75 mm^2^ (1.0 mm × 0.75 mm) of the BHK cell monolayer, was taken and the areas (number of pixels) occupied by the fused (syncytial) cells were measured with the aid of a graphics software, NIH ImageJ ver.1.45s. The percentage of the syncytial areas to the total area (0.75 mm^2^ = 3,871,488 pixels) was regarded as the fusion index. Randomly taken 10 photographs in one well (9 cm^2^) were measured for each sample and the average fusion index (%) and standard deviation were determined. Statistical significance of the data was evaluated with one-way ANOVA (Games-Howell method) using the SPSS Ver. 24 software (IBM). Differences were considered statistically significant if *p* < 0.05.

### Western Blot

Subconfluent BHK cells (ca. 3.0 × 10^6^) in one well of six-well culture plate were transfected with 2 μg/well of the pcDL-SRα expression vector encoding each F protein as described above. After 12 h of incubation at 37°C, the cells were lysed on ice for 30 min with 400μl/well of lysis buffer (50 mM HEPES [pH 7.3], 10 mM lauryl maltoside, 1 mM phenylmethylsulfonyl fluoride, 100 mM NaCl). An aliquot (15 μl) of each cell lysate was subjected to sodium dodecyl sulfate–polyacrylamide gel electrophoresis (SDS-PAGE) under reducing conditions and the separated proteins were electroblotted to Protran BA 85 nitrocellulose membrane (GE Healthcare Life Sciences) using a semi-dry transfer system (Bio-Rad). The membrane was treated with MAb 1D1 or anti-β-actin monoclonal antibody, followed by successive treatment with biotinylated anti-mouse IgG horse immunoglobulin and streptavidin/biotin/peroxidase complex (Vectastain ABC Kit, Vector Laboratories). The F protein bands were then visualized by enhanced chemiluminescence (ECL) using the Western Blotting Luminol Reagent (Santa Cruz Biotechnology), followed by exposure to X-ray film (Konica Minolta, Tokyo, Japan).

### Cell-Surface Biotinylation and Immunoprecipitation

Subconfluent BHK cells (ca. 3.0 × 10^6^) in one well of a six-well culture plate was transfected with 2.0 μg/well of the pcDL-SRα expression vector encoding each protein. After 12 h or 24 h of incubation at 37°C, the cells were treated with 0.3 mg/ml of Sulfo-NHS-LC-Biotin (Thermo Scientific) in PBS supplemented with 0.1 mM CaCl_2_ and 1 mM MgCl_2_ at 23°C for 30 min and lysed on ice with 500 μl/well of lysis buffer: Sulfo-NHS-LC-Biotin is a membrane impermeable reagent that biotinylates the primary amines on the cell surface (Staros, 1982). The biotinylated cell-surface proteins in 150 μl of the cell lysates were then immunoprecipitated with monoclonal antibodies specific for either the HPIV2 HN protein (MAb 173-1A) or the SV41HN protein (MAb 127A-1), or with the rabbit antiserum specific for PIV5 F_2_. The biotinylated and immuneprecipitated proteins were subjected to SDS-PAGE under reducing or non-reducing conditions and the separated proteins were electroblotted to nitrocellulose membrane, treated with streptavidin-biotin-peroxidase complex, and detected by ECL as described above. The intensity of the HN or the F protein band was quantified with the aid of a graphics software, NIH ImageJ ver.1.45s, and relative surface localization level was estimated.

### Immunofluorescent Staining

Subconfluent BHK cells (ca. 3.0 × 10^6^) grown on a glass coverslip in one well of a six-well culture plate were transfected with 2.0 μg/well of the pcDL-SRα expression vector encoding each HN protein. After 24 h of incubation at 37°C, the cells were fixed with 4% paraformaldehyde in PBS, washed three times with PBS, and permeabilized or not permeabilized with 0.1% Triton X-100 in PBS. For immunofluorescent staining, the cells were treated with MAb 173-1A or MAb 127A-1, and then with Alexa-Fluor 488-conjugated goat anti-mouse IgG H&L (Abcam). The results were observed by using a fluorescence microscope (Olympus, Tokyo, Japan).

### Molecular Modeling

Molecular modeling of the HN and F proteins was performed on the automated comparative protein modeling server, SWISS-MODEL^1^, by using the crystal structures of the PIV5 (WR) F protein (PDB ID: 4WSG) and the PIV5 (W3A) HN protein (PDB ID: 4JF7) as the templates, respectively. The crystal structure of the PIV5 (W3A) HN head domain (PDB ID: 1Z4Z) was also used as a template. The downloaded PDB files were analyzed with the aid of a graphics software, Waals (Altif Laboratories, Tokyo, Japan). The quality of the model structures was assessed with QMEAN on SWISS-MODEL (**Supplementary Figure S3**).

## Results

### The F Stalk Domain Modifies the HN Protein Specificity

**FIGURE 1:**
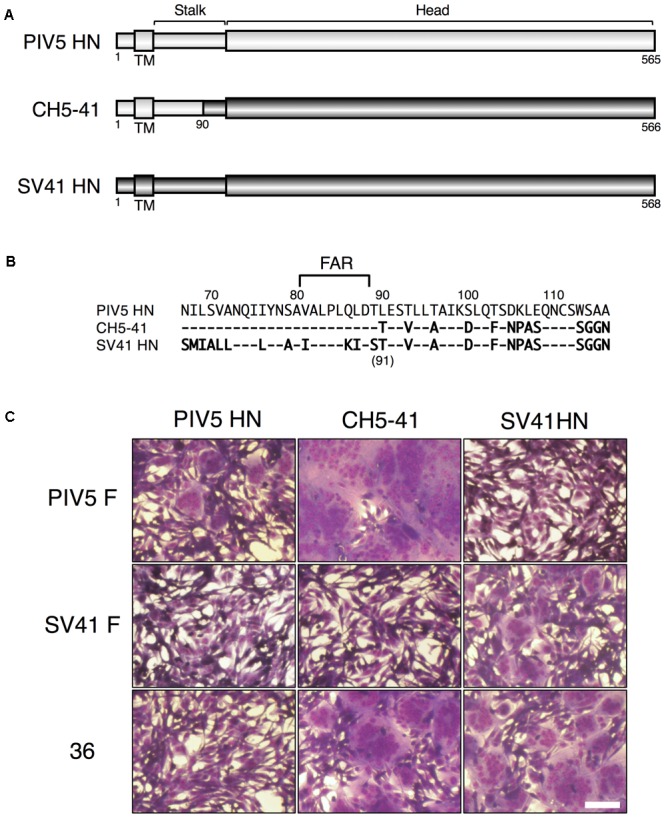
The HN protein specificity of the SV41 F-like chimeric F protein no. 36 is not identical to that of the SV41 F protein. **(A)** Schematic diagrams of the PIV5 HN protein, SV41 HN protein, and the chimeric HN protein, CH5-41. Black cylinders represent regions derived from the PIV5 HN protein, while gray cylinders represent regions derived from the SV41 HN protein. **(B)** Amino acid sequence alignment of the stalk domains of the PIV5 HN protein, CH5-41, and the SV41 HN protein. Dashes in the CH5-41 and SV41 HN protein sequences indicate the amino acids identical to the PIV5 HN counterparts. **(C)** Syncytia induced by co-expression of HN and F proteins. Subconfluent BHK cells grown in six-well culture plates were transfected with a mixture of 1 μg/well of the pcDL-SRα expression vector encoding each HN protein and 2 μg/well of the pcDL-SRα expression vector encoding each F protein. After 12 h of incubation at 37°C, the cells were fixed with 4% paraformaldehyde in PBS, treated with methanol, and stained with Giemsa’s solution. Bar, 100 μm.

**FIGURE 2:**
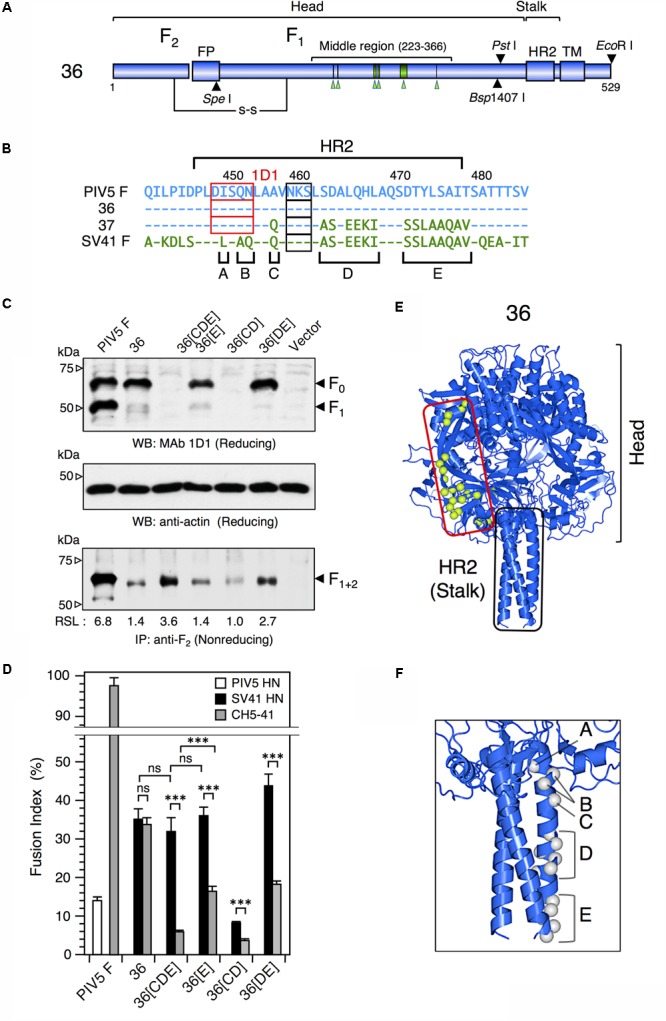
The F stalk domain modifies the HN protein specificity. **(A)** Schematic diagrams of the chimeric F protein, no. 36. Blue cylinders represent regions derived from the PIV5 F protein. Six green arrowheads below the middle region indicate the positions of SV41 F-derived 21 amino acids. Filled triangles indicate the positions of restriction enzyme sites in the no. 36-encoding cDNA. TM, transmembrane domain; FP, fusion peptide; HR2; heptad repeat 2. **(B)** Amino acid sequence alignment of the HR2 domains of the PIV5 F protein, no. 36, no. 37, and the SV41 F protein. Dashes in the no. 36, no. 37, and the SV41 F sequences indicate the amino acids identical to the PIV5 F counterparts. The amino acids that are not shared by no. 36 (or the PIV5 F protein) and the SV41 F protein are grouped into five clusters (A, B, C, D, and E). Potential *N*-glycosylation sites are boxed. The core epitopes for MAb 1D1 are shown as red boxes. **(C)** Detection of the F proteins in the plasmid-transfected cells. Subconfluent BHK cells in six-well culture plates were transfected with 2 μg/well of the pcDL-SRα expression vector encoding each F protein. (Upper and middle panels) In order to detect total F proteins expressed in the transfected cells, cell lysates were lysed at 12 h post transfection and subjected to SDS-PAGE under reducing conditions, followed by Western blot with MAb 1D1 or anti-β-actin monoclonal antibody as described in the section “Materials and Methods.” (Lower panel) In order to detect the F proteins expressed on the cell surface, the transfected cells were biotinylated at 12 h post transfection. The cell lysates were then subjected to immunoprecipitation with anti-PIV5 F_2_ rabbit serum and the precipitates were separated by SDS-PAGE under non-reducing conditions. The separated proteins were electroblotted to nitrocellulose membrane, treated with streptavidin-biotin-peroxidase complex, and detected by ECL as described in the section “Materials and Methods.” The biotinylated F protein bands represent the cleaved form, F_1+2_, as reported previously (Tsurudome et al., 2006, 2013). The relative surface-localization (RSL) level of F_1+2_ is presented below each lane. Vector: pcDL-SRα expression vector used as the negative control. **(D)** HN protein specificity of the F proteins. Subconfluent BHK cells in six-well culture plates were transfected with a mixture of 2 μg/well of the pcDL-SRα expression vector encoding each F protein and 1 μg/well of the pcDL-SRα expression vector encoding each HN protein. After 12 h of incubation, the cells were fixed with 4% paraformaldehyde and the average fusion index was determined as described in the section “Materials and Methods”; error bars indicate standard deviation. The representative data of more than three independent experiments are shown. The fusion indices are not normalized to the cell surface-localization levels; the normalized data are shown in **Supplementary Figure S4A**. The statistical significance was evaluated by one-way ANOVA as described in the section “Materials and Methods” (^∗∗∗^*p* < 0.01, *n* = 10). ns, not significant. **(E)** Predicted three-dimensional structure of no. 36. The side view of the no. 36 trimer is drawn as a ribbon model on the basis of the crystal structure of PIV5(WR) F protein (PDB ID: 4WSG). No. 36 is a chimeric PIV5 F protein which harbors SV41 F-derived 21 amino acids (indicated as light green balls) in the middle region of the head domain; those in only one protomer are shown for clarity. **(F)** Predicted three-dimensional structure of the HR2 region of no. 36. Gray balls indicate the positions of unconserved amino acids in the five clusters (A, B, C, D, and E) shown in **Figure 2B**; those in only one protomer are shown for clarity. The most carboxy-terminal residue in the model structure is A477 in the cluster E. The amino acid sequence identity between the ectodomains (amino-terminal 477 residues) of no. 36 and the PIV5 F protein is 96.0%.

**FIGURE 3:**
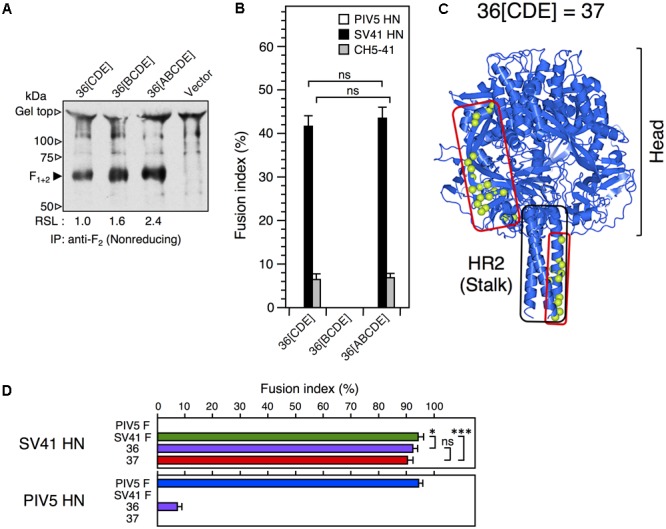
No. 37 is a more SV41 F-like protein than no. 36. **(A)** Detection of the F proteins in the plasmid-transfected cells. Subconfluent BHK cells in six-well culture plates were transfected with 2 μg/well of F expression vector. The biotinylated F proteins expressed on the cell surface were detected by immunoprecipitation with anti-PIV5 F_2_ rabbit serum as described in the legend for **Figure 1C**. Vector: pcDL-SRα expression vector used as the negative control. RSL, relative surface localization. **(B)** HN protein specificity of the F proteins. Subconfluent BHK cells in six-well culture plates were transfected with 2 μg/well of the pcDL-SRα expression vector encoding each F protein together with 1 μg/well of the pcDL-SRα expression vector encoding each HN protein. After 12 h of incubation, the cells were fixed with 4% paraformaldehyde and the average fusion index was determined as described in the section “Materials and Methods”; error bars indicate standard deviation. The representative data of more than three independent experiments are shown. The fusion indices are not normalized to the cell surface-localization levels; the normalized data are shown in **Supplementary Figure S5B**. The statistical significance was evaluated by one-way ANOVA as described in the section “Materials and Methods”. ns, not significant. **(C)** Predicted three-dimensional structure of no. 37. The side view of the no. 37 trimer is drawn as a ribbon model on the basis of the crystal structure of PIV5(WR) F protein (PDB ID: 4WSG). The amino acid sequence identify between the ectodomains (amino-terminal 477 residues) of no. 37 and the PIV5 F protein is 93.6%. Light green balls surrounded by red lines indicate the positions of SV41 F-derived 36 amino acids; those in only one protomer are shown for clarity. **(D)** F protein specificity of the HN proteins. Subconfluent BHK cells in six-well culture plates were transfected with a mixture of 0.2 μg/well of each HN-encoding vector and 2 μg/well of each F-encoding vector. The transfected cells were fixed with 4% paraformaldehyde at 24 h post transfection and the average fusion index was determined as described in the section “Materials and Methods”; error bars indicate standard deviation. The representative data of more than three independent experiments are shown. The fusion indices are not normalized to the cell surface-localization levels. The statistical significance was evaluated by one-way ANOVA as described in the section “Materials and Methods” (^∗^*p* < 0.05, ^∗∗∗^*p* < 0.01, *n* = 10). ns, not significant.

We reported previously that the PIV5 F protein can be converted to an SV41 F-like protein by replacing 21 amino acids in its head domain with those of the SV41 F protein (Tsurudome et al., 2013). Indeed, the resulting chimeric protein, no. 36, was activated by the SV41 HN protein and induced prominent cell–cell fusion in BHK cells at 12 h after co-transfection, but not by the PIV5 HN protein (**Figure 1C**). In order to ascertain in the current study whether this SV41 F-like chimeric protein really has an HN protein specificity that is identical to that of the SV41 F protein, we employed a chimeric HN protein, CH5-41, whose ectodomain is composed of PIV5 HN-derived stalk domain and SV41 HN-derived head domain (**Figures 1A,B**), because it activated the PIV5 F protein seven-times more efficiently compared to the PIV5 HN protein at 12 h after co-transfection while it could not activate the SV41 F protein at all (Tsurudome et al., 2011). Contrary to our expectation, however, CH5-41 has proven to be able to activate no. 36 very efficiently (**Figure 1C**), indicating that the HN protein specificity of no. 36 is not identical to that of the SV41 F protein and suggesting that this chimeric PIV5 F protein needs more SV41 F-derived amino acids in order to not be activated by CH5-41. This reminded us of our previous finding that the heptad repeat 2 (HR2) region of the parainfluenza virus F protein affects the HN protein specificity (Tsurudome et al., 1998). We then performed chimeric analysis of the HR2 region of no. 36 (**Figures 2A,E**), which harbored 17 amino acids [grouped into five clusters (A, B, C, D, and E) for convenience’s sake] that were not shared with the SV41 F protein (**Figures 2B,F**). First, we replaced the carboxy-terminal three clusters (C, D, and E) of no. 36 with those of the SV41 F protein since these clusters were downstream of the epitope for the detecting antibody, MAb 1D1; the resulting chimera no. 36[CDE] was expressed on the transfected cell surface more efficiently than no. 36 as detected by anti-F_2_ antiserum (**Figure 2C**, lower panel). We found that CH5-41 activated no. 36[CDE] far less efficiently than it activated no. 36 when judged at 12 h post transfection, whereas the SV41 HN protein equally activated these chimeric F proteins (**Figure 2D**), indicating that the HR2 region is indeed involved in determining the HN protein specificity. Secondly, we replaced stepwise the SV41 F-derived three clusters of no. 36[CDE] with the PIV5 F counterparts. As shown in **Figure 2D** and **Supplementary Figure S4B**, the resulting chimeras no. 36[E], no. 36[CD], and no. 36[DE] showed intermediate HN protein specificities between those of no. 36 and no. 36[CDE]. Intriguingly, no. 36[CDE], and no. 36[CD] could not be detected by MAb 1D1 in the Western blot despite that these chimeras retained the core epitope for this antibody (**Figure 2C**, upper panel), suggesting that replacement of cluster C (or A456) had somehow affected the conformation of the core epitope in the electroblotted form. Thirdly, replacement of all five clusters resulted in the chimera no. 36[ABCDE] which showed an HN protein specificity indistinguishable from that of the chimera no. 36[CDE] (**Figure 3B**) and lower efficiency of fusion induction compared to 36[CDE] (**Supplementary Figure S5B**). On the other hand, replacement of four clusters resulted in the chimera no. 36[BCDE] which could be activated by neither of the HN proteins (**Figure 3B**), despite that it was efficiently cleaved and expressed on the cell surface even more efficiently compared to no. 36[CDE] (**Figure 3A** and **Supplementary Figure S5A**). Finally, we concluded that replacement of 15 amino acids in the three clusters (C, D, and E) of the HR2 regions were enough to make no. 36 to a more SV41 F-like chimeric protein as represented by no. 36[CDE], which was renamed as no. 37 for convenience’s sake (**Figure 3C**). Being consistent with this conclusion, we found that the PIV5 HN protein was able to activate no. 36 though weakly but not no. 37 at all when judged at 24 h post transfection, while the SV41 HN protein equally activated these chimeric F proteins (**Figure 3D**). It should be pointed out again, in this context, that the F-triggering activity of the PIV5 HN protein toward no. 36 was not detectable when judged at 12 h post transfection (**Figure 1C**) (Tsurudome et al., 2013).

### Combination of the SV41 HN-Derived Region III With Specified SV41 HN-Derived Amino Acids in the Region II Is Important for Exhibiting the SV41 HN-Like F Protein Specificity

**FIGURE 4:**
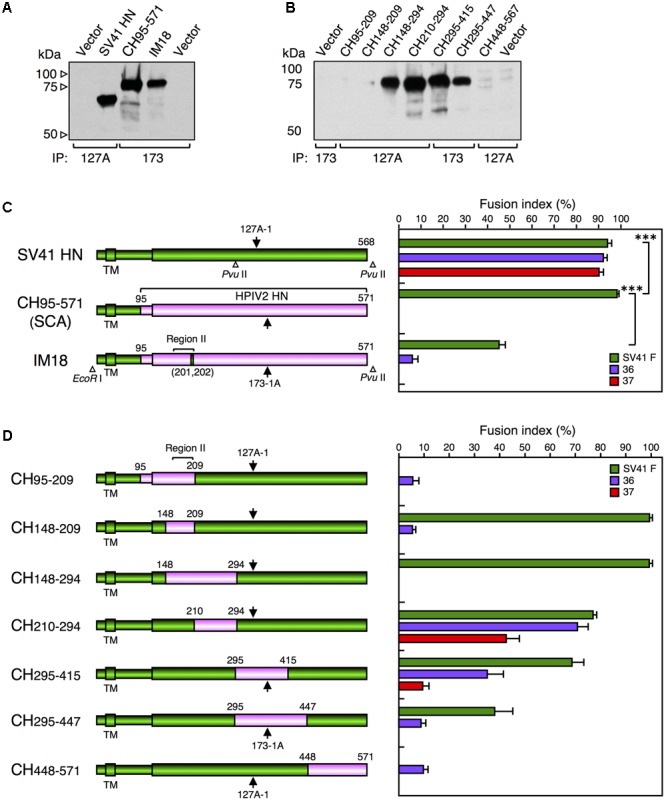
Middle region of the SV41 HN protein is not required for activating the SV41 F-like chimeric proteins. **(A,B)** Detection of cell surface-localized HN proteins. Subconfluent BHK cells in six-well culture plates were transfected with 2 μg/well of the pcDL-SRα expression vector encoding each HN protein. At 24 h post transfection, the transfected cells were biotinylated, the cell lysates were subjected to immunoprecipitation (IP) with MAb 173-1A (173) or MAb 127A-1 (127A), the precipitates were subjected to SDS-PAGE under reducing conditions, and the HN protein bands were detected by ECL as described in the section “Materials and Methods.” Vector: pcDL-SRα expression vector used as the negative control. The SV41 HN protein migrates much faster compared to CH95-571 and IM18 as reported previously (Tsurudome et al., 2015), presumably reflecting the remarkable difference in the number of potential *N*-glycosylation sites between the HN proteins of SV41 and HPIV2 (Tsurudome et al., 1990): the SV41 HN protein (568 aa) migrates as a 67-kDa protein (Tsurudome et al., 1989) while the HPIV2 HN protein (571 aa) migrates as an 82-kDa protein (Ito et al., 1987; Tsurudome et al., 1990). **(C,D)** F protein specificity of the HN proteins. The average fusion index was determined at 24 h post transfection as described in the legend for **Figure 2D**; error bars indicate standard deviation. The representative data of more than three independent experiments are shown. The fusion indices are not normalized to the cell surface-localization levels which could not be determined due to the use two detection antibodies. The statistical significance was evaluated by one-way ANOVA as described in the section “Materials and Methods” (^∗∗∗^*p* < 0.01, *n* = 10). ns, not significant. Open triangles indicate the positions of restriction enzyme sites in the cDNA encoding the SV41 HN protein or IM-18. Arrows indicate the positions of epitopes for the two anti-HN monoclonal antibodies. The numbers above each cylinder denote the residue numbers of the HPIV2 HN fragments that had replaced the corresponding part of the SV41 HN protein.

**FIGURE 5:**
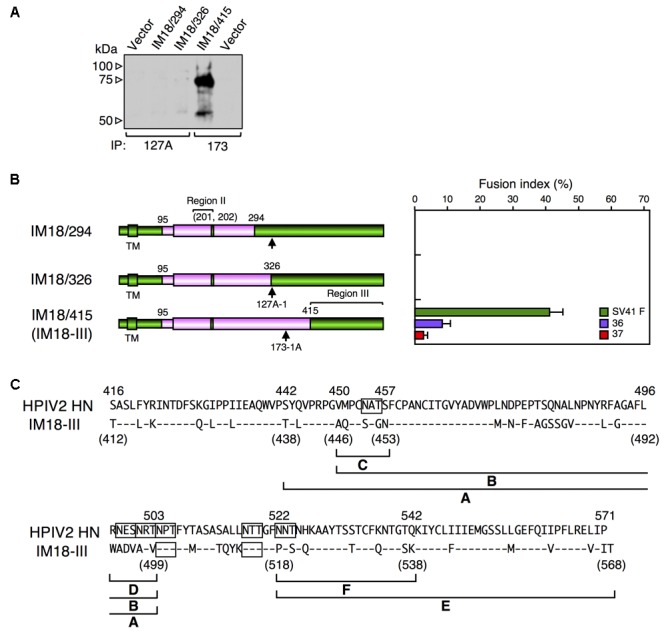
Combination of the SV41 HN-derived region III with specified SV41 HN-derived amino acids in the Region II is important for exhibiting the SV41 HN-like F protein specificity. **(A)** Detection of cell surface-localized HN proteins. The cell surface-localized HN proteins were detected as described in the legend for **Figures 4A,B**. Vector: pcDL-SRα expression vector used as the negative control. **(B)** F protein specificity of the HN proteins. The average fusion index was determined at 24 h post transfection as described in the legend for **Figure 3D**; error bars indicate standard deviation. The residue numbers of SV41 HN-derived amino acids in the region II was indicated in the parenthesis. The representative data of more than three independent experiments are shown. The fusion indices are not normalized to the cell surface-localization levels which could not be determined due to the use two detection antibodies. **(C)** Amino acid sequence alignment of the region III of the HPIV2 HN protein and IM18-III. Dashes in the IM18-III sequence indicate the amino acids identical to the HPIV2 HN counterparts. Potential *N*-glycosylation sites are boxed. The positions of the SV41 HN-derived segments (shown in **Figures 6C,D**) are indicated below the IM18-III sequence.

We reported previously that the SV41 HN-like chimeric protein, CH95-571, whose ectodomain is composed of SV41 HN-derived stalk domain and HPIV2 HN-derived head domain, can activate the SV41 F protein but not the SV41 F-like chimeric PIV5 F protein, no. 36 (Tsurudome et al., 2015). However, we also found that replacement of specified amino acids of the region II in the CH95-571 head domain with the SV41 HN counterparts resulted in a number of chimeras that can activate both of the F proteins (Tsurudome et al., 2015). For example, the chimera IM18 which harbored SV41 HN-derived two amino acids at positions 201 and 202 in the region II in addition to the SV41 HN-derived stalk domain, activated no. 36 despite activating the SV41 F protein less efficiently compared to CH95-571 (**Figure 4C**); CH95-571 was also called as SCA for convenience’s sake (Tsurudome et al., 2015). However, the triggering activity of IM18 toward no. 36 was very weak and that toward no. 37 was under detection level (**Figure 4C**). We then intended to find a region in the SV41 HN protein, other than the stalk domain and the region II, that would be required for triggering these SV41 F-like chimeric proteins as well as the SV41 F protein. To this end, we employed seven chimeric HN proteins of SV41 and HPIV2 (**Figures 4B,D** and **Supplementary Figure S6**) and examined their F protein specificity. The result showed that two chimeras, CH210-294 and CH295-415, were able to activate all of the F proteins (**Figure 4D**). They harbored non-overlapping HPIV2 HN-derived middle regions, each of which was sandwiched between two SV41 HN-derived regions: the amino-terminal and carboxy-terminal regions with different lengths. Since their SV41 HN-derived amino-terminal regions included both the stalk domain and the region II, we anticipated that other parts of the SV41 HN-derived amino-terminal regions of CH210-294 and CH295-415 might not be important. To test this possibility, we created three chimeras in which the HPIV2 HN-derived carboxy-terminal region of IM18 was replaced with the SV41 HN-derived carboxy-terminal regions of CH210-294 and CH295-415, resulting in IM18/294 and IM18/415, respectively (**Figure 5B**). The result showed that IM18/415 succeeded in activating all of the F proteins while IM18/294 failed to activate any one of the F proteins most likely due to its unsuccessful cell surface localization (**Figure 5A** and **Supplementary Figure S7**). We then created an additional chimera IM18/326, which was an intermediate between IM18/294 and IM18/415. However, this chimera was not detected neither on the surface or inside of the cell (**Figure 5A** and **Supplementary Figure S7**) and activated none of the F proteins (**Figure 5B**). We thus adopted IM18/415 as the object for further investigation; its carboxyl-terminal 156 amino acid-region derived from the SV41 HN protein was named as region III and, accordingly, IM18/415 was renamed as IM18-III (**Figures 5B,C**).

### SV41 HN-Derived Segment F Is Important for Exhibiting the SV41 HN-Like F Protein Specificity

**FIGURE 6:**
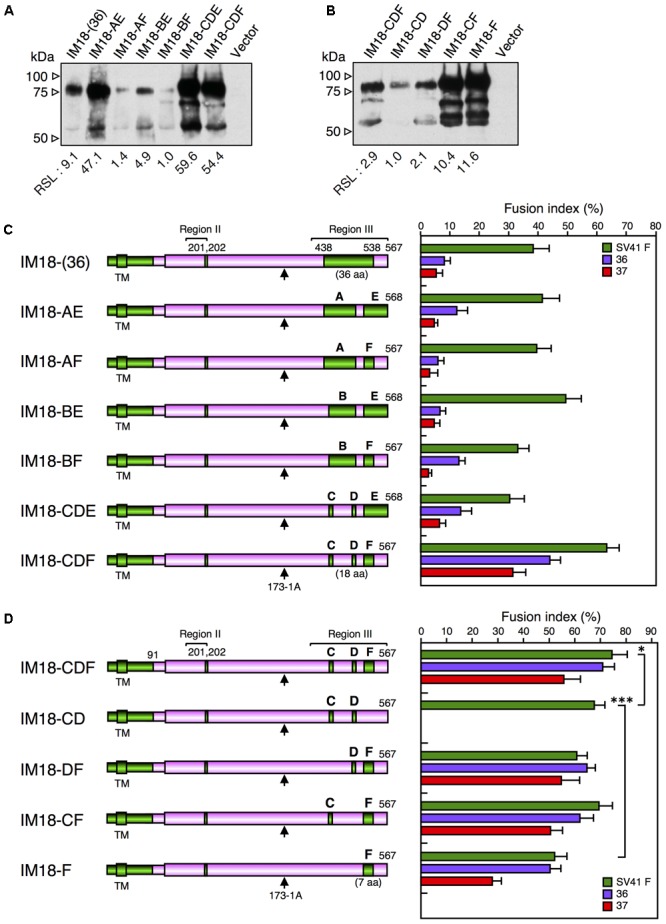
SV41 HN-derived segment F is important for exhibiting the SV41 HN-like F protein specificity. **(A,B)** Detection of cell surface-localized HN proteins. The cell surface-localized HN proteins were detected as described in the legend for **Figures 4A,B**. Vector: pcDL-SRα expression vector used as the negative control. RSL, relative surface localization. **(C,D)** F protein specificity of the HN proteins. The average fusion index was determined at 24 h post transfection as described in the legend for **Figure 2D**; error bars indicate standard deviation. The representative data of more than three independent experiments are shown. The fusion indices are not normalized to the cell surface-localization levels; the normalized data are shown in **Supplementary Figures S8A,B**. The statistical significance was evaluated by one-way ANOVA as described in the section “Materials and Methods” (^∗^*p* < 0.05, ^∗∗^*p* < 0.01, *n* = 10). Capitals above the cylinders indicate the SV41 HN-derived segments in the region III, which correspond to those shown in **Figure 5C**. Arrows indicate the positions of MAb 173-1A epitopes. The numbers of SV41 HN-derived amino acids in the region III were shown in the parentheses.

As described above, IM18-III was able to activate the SV41 F-like chimeric F proteins, no. 36 and no. 37, in addition to the SV41 F protein, though its F triggering activity toward the SV41 F-like chimeric F proteins was very low compared to that of the SV41HN protein (**Figures 4C, 5B**). Next, since the region III of IM18-III contained 48 amino acids that were not shared with the HPIV2 HN protein (**Figure 5C**), we intended to narrow down these amino acids. To this end, we replaced stepwise the SV41 HN-derived amino acids from both sides of the region III of IM18-III with those of the HPIV2 HN protein. Finally, as represented by IM18-(36) in **Figure 6C**, the number of the SV41 HN-derived amino acids in this region could be reduced from 48 to 36 without greatly affecting the F protein specificity of IM18-III suggesting that SV41 HN-derived 12 amino acids (upstream of T438 and downstream of K538) of the region III (**Figure 5C**) might not be required for IM18-III to exhibit the SV41 HN-like F protein specificity. Then, we further replaced the SV41 HN-derived amino acids in the region III of the chimera IM18-III with the HPIV2 HN counterparts as shown in **Figure 6C**. Among the six newly created chimeras, IM18-CDF proved to activate all three F proteins much more efficiently compared to IM18-(36), indicating that SV41 HN-derived 36 amino acids (aa 438–538) in the region III of IM18-(36) can be reduced to 18, which are distributed to three segments C, D, and F (**Figures 5C, 6C**). On the other hand, the chimeras IM18-AE, IM18-AF, IM18-BE, IM18-BF, and IM18-CDE activated all three F proteins at similar levels to that of IM18-(36) (**Figure 6C**), although their cell surface-localization levels varied tremendously (**Figure 6A** and **Supplementary Figure S9**) and the efficiency of fusion promotion largely differed from each other **(Supplementary Figure S8A**). To evaluate possible role of each segments of IM18-CDF in defining the F protein specificity, we created four chimeric HN proteins and tested their F protein specificity (**Figures 6B,D** and **Supplementary Figure S10**). The result indicated that presence of the SV41 HN-derived segment F was sufficient for IM18-CDE to exhibit the SV41 HN-like F protein specificity as represented by IM18-F (**Figure 6D**). Interestingly, since the efficiency of fusion promotion by IM18-F was significantly lower compared to IM18-DF (**Supplementary Figure S8B**), the presence of the segment D seemed helpful to the segment F in order to fully function. It was noteworthy, in this context, that the chimera IM18-CD, which harbored two segments (C and D) in the region III, failed to activate no. 36 and no. 37 whereas it activated the SV41 F protein far more efficiently than the IM18-F did (**Figure 6D** and **Supplementary Figure S8B**), indicating that these two segments in themselves do not contribute to the SV41 HN-like phenotype of IM18-CDE.

### SV41 HN-Derived K538 Is Indispensable for Exhibiting the SV41 HN-Like F Protein Specificity

**FIGURE 7:**
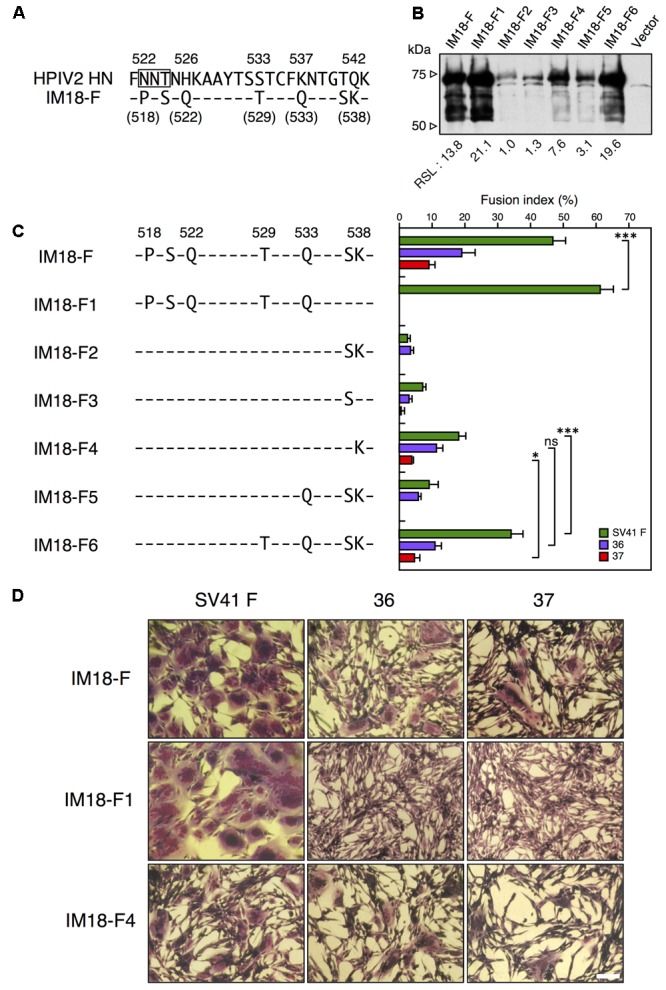
SV41 HN-derived K538 is indispensable for exhibiting the SV41 HN-like F protein specificity. **(A)** Amino acid sequence alignment of the segment F of the chimera IM18-F and the HPIV2 HN protein. Dashes in the IM18-F sequence indicate the amino acids identical to the HPIV2 HN counterparts. **(B)** Detection of cell surface-localized HN proteins. The cell surface-localized HN proteins were detected as described in the legend for **Figures 4A,B**. Vector: pcDL-SRα expression vector used as the negative control. RSL, relative surface localization. **(C)** F protein specificity of the HN proteins. The SV41 HN-derived amino acids in the segment F of the chimera IM18-F were replaced stepwise with the HPIV2 HN counterparts, resulting in six chimeric HN proteins. The average fusion index was determined at 24 h post transfection as described in the legend for **Figure 2D**; error bars indicate standard deviation. The representative data of more than three independent experiments are shown. The fusion indices are not normalized to the cell surface-localization levels; the normalized data are shown in **Supplementary Figure S8C**. The statistical significance was evaluated by one-way ANOVA as described in the section “Materials and Methods” (^∗^*p* < 0.05, ^∗∗∗^*p* < 0.01, *n* = 10). ns, not significant. **(D)** Syncytia induced by co-expression of HN and F proteins. Subconfluent BHK cells grown in six-well culture plates were transfected with a mixture of 0.2 μg/well of the pcDL-SRα expression vector encoding each HN protein and 2 μg/well of the pcDL-SRα expression vector encoding each F protein. After 24 h of incubation at 37°C, the cells were fixed with 4% paraformaldehyde in PBS, treated with methanol, and stained with Giemsa’s solution. Bar, 100 μm.

Since the segment F of IM18-F harbored SV41 HN-derived seven amino acids (**Figures 5C, 7A**), we decided to evaluate their individual roles in determining the F protein specificity. To begin with, we tried to ascertain the possible importance of S537 and K538 at the carboxy-terminus of the segment F (**Figure 7A**). Accordingly, we created chimera IM18-F1, in which these two amino acids had been replaced with the HPIV2 HN counterparts (**Figure 7C**). Interestingly, M18-F1 failed to activate no. 36 and no. 37 whereas it activated the SV41 F protein even more prominently compared to IM18-F (**Figures 7C,D**), indicating that the SV41 HN-derived S537 and/or K538 are indispensable for IM18-F to exhibit the SV41 HN-like F protein specificity while the other five (P518, S520, T229, and Q533) are not required. The importance of K538 was confirmed by IM18-F4, which harbored K538 in its segment F and activated all three F proteins though weakly (**Figures 7C,D**), while that of S537 could not be fully confirmed because the IM18-F3, which harbored S537 in its segment F, displayed very low F-triggering activity especially toward no. 37 (**Figure 7C**) and was insufficiently localized on the cell surface (**Figure 7B** and **Supplementary Figure S11**). Moreover, M18-F2, which harbored S537 and K538 in its segment F, was poorly localized on the cell-surface (**Figure 7B** and **Supplementary Figure S11**), showed extremely low F-triggering activity, and failed to activate no. 37 (**Figure 7C**). As represented by IM18-F6, however, addition of SV41 HN-derived T529 and Q533 to M18-F2 raised the cell surface-localization level and endowed it with the SV41 HN-like F protein specificity (**Figure 7C**) whereas efficiency of fusion promotion by IM18-F6 toward no. 36 was significantly low compared to IM18-F2 (**Supplementary Figure S8C**). It should be pointed out, in this context, that the F-triggering activity of IM18-F6 toward no. 36 and no. 37 was similar to that of IM18-F4 despite that IM18-F6 induced fusion with the SV41 F protein more extensively compared to IM18-F4, suggesting IM18-F4 is a more SV41-HN like protein compared to IM18-F6 and that S537 does not greatly contribute to the SV41 HN-like phenotype of IM18-F. Taken together, these results indicated that K538 is the key element of the segment F of IM18-F. Importantly, all seven chimeras shown in **Figure 7C** failed to activate the HPIV2 F protein (not shown in the figure), indicating that IM18-F, IM18-F4, and IM18-F6 are not almighty HN proteins that can activate any F proteins.

**FIGURE 8:**
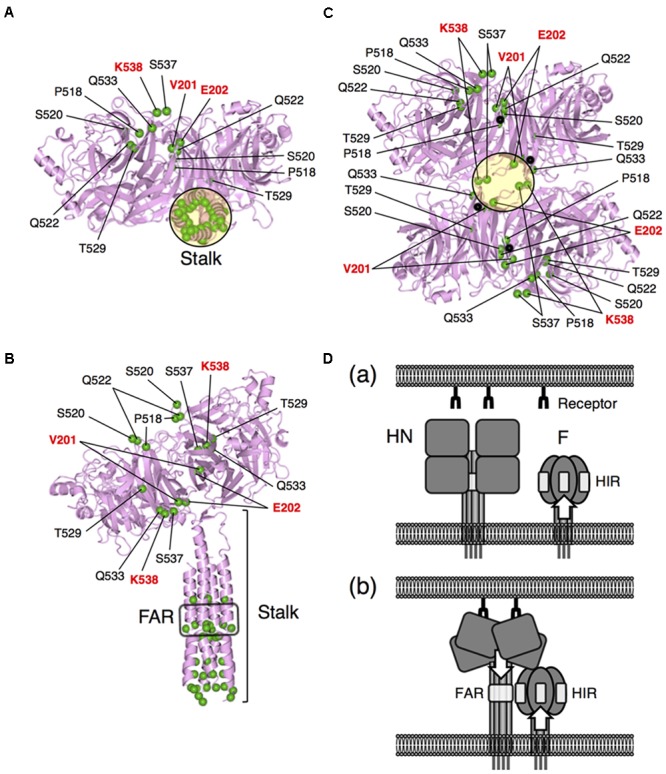
Locations of the SV41 HN-derived amino acids in the SV41 HN-like chimera IM18-F. **(A,B)** Bottom **(A)** and side **(B)** views of IM18-F in the “2-heads-up/2-heads-down” conformation. The structures were drawn as ribbon models on the basis of the crystal structure (PDB ID: 4JF7) of the PIV5 (W3A) HN protein. The most amino-terminal residue in the model structure (ectodomain) is I62 in the stalk domain. The amino acid sequence identify between the ectodomains of IM18-F and the PIV5 HN protein is 47.3%. The head domains of the protomers which are in the down conformation are not shown. The positions of α-carbons of the SV41 HN-derived amino acids are indicated as green balls; in **(A)**, only four amino acids (residue numbers in the parentheses) of the right protomer can be seem because the remaining five are hidden behind the stalk domain. FAR, F-activating region. **(C)** Bottom view of the head domain of IM18-F in the “4-heads-up” conformation. The structure was drawn as a ribbon model on the basis of the crystal structure (PDB ID: 1Z4Z) of the “receptor bound form” of PIV5 (W3A) HN head domain. The position of the stalk domain is predicted to be at the center of the tetramer and indicated as a black circle as in **(A)**. The positions of α-carbons of the SV41 HN-derived amino acids are indicated as green balls. A black ball in each protomer indicates the α-carbons of the most amino-terminal residue L119 of the model structure. The amino acid sequence identify between the head domains of IM18-F and the PIV5 HN protein is 50.8%. **(D)** A hypothesis for the HN-F interaction. **(a)** Before receptor binding, the conformation of the HN-interacting region (HIR) in the F head domain is already modified if the F stalk domain has critical mutations. **(b)** After receptor binding, the HN protein undergoes a structural transition from the 4-heads-down conformation to the 4-heads-up conformation, which allows the FAR in the HN stalk domain to be exposed and to interact with the HIR in the F head domain. The conformation of the exposed FAR can be modified by critical mutations in the HN head domain.

Finally, we concluded that, in order to convert the HPIV2 HN protein to an SV41 HN-like protein, the seven amino acids (P518, S520, Q522, T529, Q533, S537, and K538) in the segment F, as well as the stalk domain and the two amino acids (V201 and E202) in the region II, should be replaced with the SV41 HN counterparts as represented by IM18-F (**Figures 6C, 7A**); among the seven residues in the segment F, K538 seemed to play the most important role. As shown in **Figure 8C**, these critical amino acids in the region II and segment F are located at the dimer interfaces of the IM18-F4 head domain in the 4-heads-up conformation; half of them are also located at the tetramer interface just above the stalk domain (**Figures 8A–C**).

## Discussion

We reported previously that a chimeric PIV5 F protein, no. 36, which harbored SV41 F-derived 21 amino acids in the head domain, was activated by the SV41 HN protein but not by the PIV5 HN protein as judged by cell–cell fusion assay in BHK cells at 12 h post transfection (Tsurudome et al., 2013) (**Figure 1C**). However, when judged at 24 h post transfection, the PIV5 HN protein proved to be able to activate no. 36 though weakly (**Figure 3D**). We further found that replacing 15 amino acids in the HR2 domain of no. 36 with the SV41 F counterparts resulted in chimera no. 37, which was specifically activated by the SV41 HN protein but not by the PIV5 HN protein (**Figure 3D**). Thus, in order to convert the PIV5 F protein to an SV41 F-like protein, the 15 amino acids in the stalk domain as well as the 21 amino acids in the head domain should be replaced with those of the SV41 F protein (**Figure 3C**). By employing no. 37, we then performed a series of chimeric analyses of the HN protein, revealing that in order to convert the HPIV2 HN protein to an SV41 HN-like protein, at least nine amino acids (V201, E202, P518, S520, Q522, T529, Q533, S537, and K538) in the head domain should be replaced with the SV41 HN counterparts in addition to the replacement of the stalk domain (**Figures 6D, 7A**); among the nine amino acids, at least three amino acids (V201, E202, and K538) seem indispensable (**Figure 7C**).

According to a model proposed by Bose et al. (2015), the HN head domain undergoes a structural transition upon attachment from the 4-heads-down conformation to the 4-heads-up conformation, which allows otherwise hidden fusion activating region (FAR) in the HN stalk domain to interact with the HN-interacting region (HIR) in the F head domain (**Figure 8D**). The F protein then undergoes a drastic refolding that leads to membrane merger. This HN-F interaction is virus type-specific in principle and whether such HN-F interaction takes place between the glycoproteins of different viruses principally depends on the similarity in the primary structures between FAR and those between the HIR. However, we found recently that any single amino acid in the HN stalk domain cannot explain the unidirectional substitutabilities among rubulavirus HN proteins, suggesting that conformation of the HN stalk domain might be critical for defining the F protein specificity (Tsurudome et al., 2015). Since the critical nine amino acids in the head domain of the SV41 HN-like chimera IM18-F are located at or around the dimer and/or tetramer interfaces in the 4-heads-up conformation (**Figures 8A–C**), mutations of these amino acids may readily affect the quaternary structure of the HN head domain, which would then modify the quaternary structure of the HN stalk domain (**Figure 8D**). Indeed, the SV41 HN-like chimeric HN protein, SCA, which harbors SV41 HN-derived stalk domain and HPIV2 HN-derived head domain, efficiently activates the SV41 F protein but not no. 36 and no. 37 (**Figure 4C**). However, replacement of the critical nine amino acids in the SCA head domain with the SV41 HN counterparts results in the chimeric HN protein, IM18-F, that is able to activate all three F proteins (**Figure 6D**), suggesting that the conformation and/or flexibility of the FAR of the IM18-F is nearly identical to that of the SV41 HN protein while that of SCA is not. Interestingly, in this context, we found recently that attachment of FLAG sequences to the cytoplasmic tail of several HN proteins modified the F protein specificity and that headless or truncated HN proteins usually exhibited altered F protein specificity (Tsurudome et al., 2015). It is thus very likely that mutations of specified amino acids in the HN head domain somehow modify the conformations of the HN stalk domain, thereby converting the specificity for the F protein. In other words, appropriate structure of the HN head domain is critical for the HN-F interaction to take place (**Figure 8D**). We thus assume that a given rubulavirus HN protein has the potential to trigger other non-cognate rubulavirus F proteins but subtle difference in the stalk conformation determines whether it can do that or not. In all likelihood, the PIV5 HN-like chimeric protein CH5-41 can activate the SV41 F-like chimeras no. 36 and No. 37 (**Figures 2D, 3B**) due to its SV41 HN-derived head domain (**Figure 1A**).

Structural and/or functional interplay between the head and stalk domains of the HN protein has become apparent during last decade. Firstly, it has been reported that the HN stalk domain affects the oligomerization of the HN head domain (Yuan et al., 2005). Secondly, it has been reported recently that the membrane-proximal region of the HN stalk domain modulates the receptor-binding and neuraminidase activities of the HN head domain (Adu-Gyamfi et al., 2016). Reciprocally, the HN head domain stabilizes the HN stalk domain, thereby regulating the F-activating function (Adu-Gyamfi et al., 2016). Lastly, our current observation suggests a more sophisticated role of the HN head domain than has previously been thought, in which it indirectly defines the F protein specificity by regulating the conformation of the HN stalk domain. Interestingly, it has recently been reported for the Nipah virus, a member of genus *Henipavirus* in the subfamily *Paramyxovirinae* (Lamb and Parks, 2013), that both the head and stalk domains of its attachment protein G can physically interact with the F protein (Stone et al., 2016), presumably reflecting considerable difference in the mechanism of F activation between the HN and G proteins (Chang and Dutch, 2012; Aguilar et al., 2016).

On the other hand, an interplay between the head and stalk domains of the F protein has been documented in our current study for the first time, which suggested that the HIR in the F head domain should assume an appropriate conformation in order to interact with the FAR in the HN stalk domain and that alterations in the F stalk structure readily modify the conformation of the HIR. Consistent with this notion, we previously found that the HN protein specificity of the F protein cannot solely be defined by the primary sequence of the HIR (Tsurudome et al., 2011, 2013). On the other hand, an immunoglobulin-like (Ig-like) domain in the domain II of the PIV5 F head domain has been proposed to be the candidate for HIR, which protrudes from the molecule such that the fusion peptide is sequestered immediately behind the Ig-like domain (Bose et al., 2013). However, our chimeric analyses have identified another candidate HIR, which involved non-contiguous 21 amino acids in the domains I and III of the F head domain (Tsurudome et al., 2015). Such discrepancy in the location of the HIR may have arisen from the difference in the experimental design: in the former experiment, the HIR was identified as the site whose mutation abolished the HN-dependent fusion activity of the F protein while, in the latter experiment, the HIR was identified as the determinant of the HN protein specificity of fusion-competent chimeric F proteins.

Our previous and current observations have suggested that in order for the wild type HN and F proteins to interact with each other, the HN stalk domain should assume a fusion-competent conformation which depends on appropriate structure of the HN head domain while the F head domain should assume a fusion-competent conformation which depends on appropriate structure of the F stalk domain. This notion suggests that there has been a functional constraint on the overall structure of the parainfluenza virus glycoproteins in order to mediate membrane fusion. Such limitation may allow them to mutate as long as the intramolecular interplay between the head and stalk domains is not affected. Hence, during evolution, the HN head domain should have had to maintain this property as well as the receptor-binding and receptor-destroying activities.

## Author Contributions

MT and JO created the recombinant plasmids and performed cell–cell fusion assay. MT performed the protein expression experiments, molecular modeling, and wrote the manuscript. MI, MN, and TN analyzed the nucleotide sequences of the recombinant plasmids and performed the molecular modeling.

## Conflict of Interest Statement

The authors declare that the research was conducted in the absence of any commercial or financial relationships that could be construed as a potential conflict of interest.

## Footnotes

1 http://swissmodel.expasy.org

## Supplementary Material

FIGURE S1 Nucleotide sequences of the oligonucleotide primers used for creation of chimeric HN proteins. **(A)** Nucleotide sequences of the oligonucleotide primers which were used for inserting respective PCR fragment into the pcDL-SRa expression vector encoding the chimeric HN protein, IM18, by the In-Fusion ligation method described in the section “Materials and Methods.” Restriction enzyme sites are underlined. **(B)** Nucleotide sequences of the oligonucleotide primers which were used for introducing chimeric junctions or mutations by PCR. For every primer, a primer with the complementary sequence was employed in order to connect the PCR fragments.

FIGURE S2 Nucleotide sequences of the oligonucleotide primers used for creation of chimeric F proteins. **(A)** Nucleotide sequences of the oligonucleotide primers which were used for inserting respective PCR fragment into the pcDL-SRa expression vector encoding the chimeric F protein, no. 36, by ligation. Restriction enzyme sites are underlined. **(B)** Nucleotide sequences of the oligonucleotide primers which were used for introducing chimeric junctions or mutations by PCR. For every primer, a primer with its complementary sequence was employed in order to connect the PCR fragments.

FIGURE S3 QMEAN scores of the model structures. QMEAN4 scores of the chimeras **(A)** and their templates **(B)** were calculated on a Web site (SWISS-MODEL). The amino acid sequence identity between each chimera and its template was also presented in **(A)**.

FIGURE S4 The F stalk domain modifies the HN protein specificity. **(A)** Efficiency of fusion induction by chimeric F proteins. The fusion indices given by the chimeric F proteins shown in **Figure 2D** are normalized to their cell surface-localization levels. The statistical significance was evaluated by one-way ANOVA as described in the section “Materials and Methods” (^∗∗∗^*p* < 0.01, *n* = 10). ns, not significant. **(B)** HN protein specificity of the F proteins. For each chimeric F protein used in **Figure 2D**, the fusion index given with CH5-41 was normalized to that given with the SV41 HN protein. The statistical significance was evaluated by one-way ANOVA as described in the section “Materials and Methods” (^∗∗∗^*p* < 0.01, *n* = 10). ns, not significant; NA, not applicable.

FIGURE S5 No. 37 is a more SV41 F-like protein than no. 36. **(A)** Detection of the F proteins in the plasmid-transfected cells. (Upper panel) long-exposed image of the data presented in **Figure 3A**; the cleaved form (F_1+2_) and unclevaed form (F_0_) are considered to comigrate with each other under non-reducing conditions. (Lower panel) under reducing conditions, the cleaved form (F_1_) nearly comigrates with an unidentified cellular protein (ca. 50 kDa), which have been precipitated by the ant-PIV5 F_2_ polyclonal antibody. In all likelihood, this 50 kDa-protein migrates much slower than F_0_/F_1+2_ under non-reducing conditions **(A)** due to disulfide-mediated association with other unidentified cellular protein(s). In the parenthesis is indicated the predicted position of the uncleaved form, F_0_. **(B)** Efficiency of fusion induction by the F proteins. The fusion indices given by the chimeric F proteins shown in **Figure 3B** are normalized to their cell surface-localization levels. The statistical significance was evaluated by one-way ANOVA as described in the section “Materials and Methods” (^∗∗∗^*p* < 0.01, *n* = 10).

FIGURE S6 Immunofluorescent staining the chimeric HN proteins used in **Figure 4B**. Subconfluent BHK cells grown on glass coverslips in six-well culture plates were transfected with 2.0 μg/well of the pcDLSRa expression vector encoding each HN protein. After 24 h of incubation at 37°C, the cells were fixed with 4% paraformaldehyde in PBS, washed three times with PBS, and permeabilized or not permeabilized with 0.1% Triton X-100 in PBS. The HN proteins were visualized by indirect immunofluorescent staining as described in the section “Materials and Methods” by using MAb 173-1A or MAb 127A-1. Bar, 100 μm.

FIGURE S7 Immunofluorescent staining the chimeric HN proteins used in **Figure 5A**. Subconfluent BHK cells grown on glass coverslips in six-well culture plates were transfected with 2.0 μg/well of the pcDLSRa expression vector encoding each HN protein. After 24 h of incubation at 37°C, the cells were fixed with 4% paraformaldehyde and the HN proteins were visualized as described in the legend for **Supplementary Figure S6** by using MAb 173-1A or MAb 127A-1. Bar, 100 μm.

FIGURE S8 Efficiency of fusion promotion by the HN proteins. The fusion indices given by the HN proteins shown in **Figure 6C (A), Figure 6B (B)**, and **Figure 7C (C)** are normalized to their cell surface-localization levels. The statistical significance was evaluated by one-way ANOVA as described in the section “Materials and Methods” (^∗∗∗^*p* < 0.01, *n* = 10). ns, not significant.

FIGURE S9 Immunofluorescent staining the chimeric HN proteins used in **Figure 6A**. Subconfluent BHK cells grown on glass coverslips in six-well culture plates were transfected with the expression vector encoding each HN protein. After 24 h of incubation at 37°C, the cells were fixed with 4% paraformaldehyde and the HN proteins were visualized as described in the legend for **Supplementary Figure S6** by using MAb 173-1A. Bar, 100 μm.

FIGURE S10 Immunofluorescent staining the chimeric HN proteins used in **Figure 6B**. Subconfluent BHK cells grown on glass coverslips in six-well culture plates were transfected with the expression vector encoding each HN protein. After 24 h of incubation at 37°C, the cells were fixed with 4% paraformaldehyde and the HN proteins were visualized as described in the legend for **Supplementary Figure S6** by using MAb 173-1A. Bar, 100 μm.

FIGURE S11 Immunofluorescent staining the chimeric HN proteins used in **Figure 7B**. Subconfluent BHK cells grown on glass coverslips in six-well culture plates were transfected with the expression vector encoding each HN protein. A After 24 h of incubation at 37°C, the cells were fixed with 4% paraformaldehyde and the HN proteins were visualized as described in the legend for **Supplementary Figure S6** by using MAb 173-1A. Bar, 100 μm.
