# Supplementary figures and images for: The Hemagglutinin-Neuraminidase (HN) Head Domain and the Fusion (F) Protein Stalk Domain of the Parainfluenza Viruses Affect the Specificity of the HN-F Interaction
Source: Front Microbiol. 2018 Mar 13;9:391. doi: 10.3389/fmicb.2018.00391 (PMC5859044; doi:10.3389/fmicb.2018.00391)

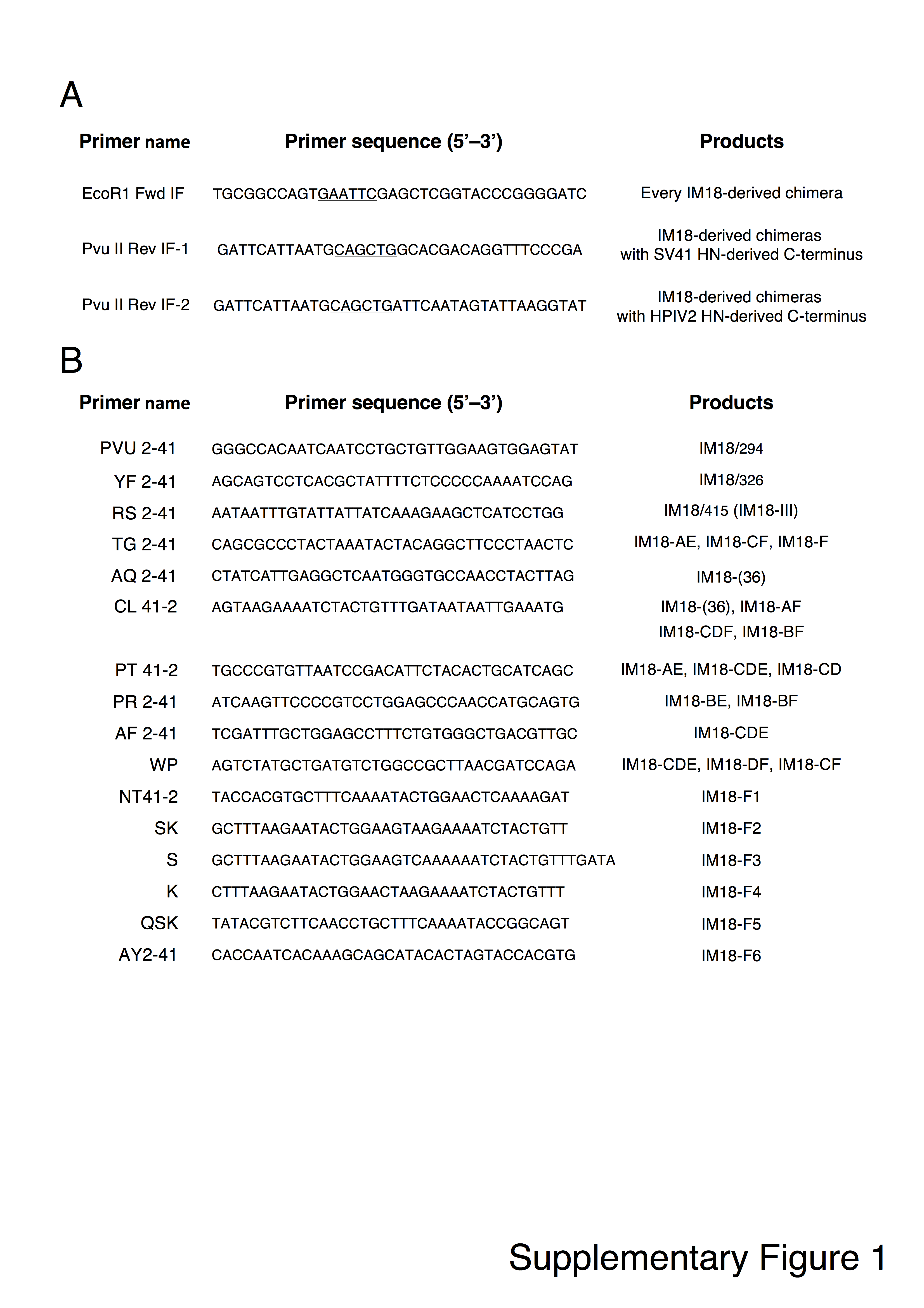

Supplement: FIGURE S1 — Nucleotide sequences of the oligonucleotide primers used for creation of chimeric HN proteins. (A) Nucleotide sequences of the oligonucleotide primers which were used for inserting respective PCR fragment into the pcDL-SRa expression vector encoding the chimeric HN protein, IM18, by the In-Fusion ligation method described in the section “Materials and Methods.” Restriction enzyme sites are underlined. (B) Nucleotide sequences of the oligonucleotide primers which were used for introducing chimeric junctions or mutations by PCR. For every primer, a primer with the complementary sequence was employed in order to connect the PCR fragments. [file Image_1.tif]

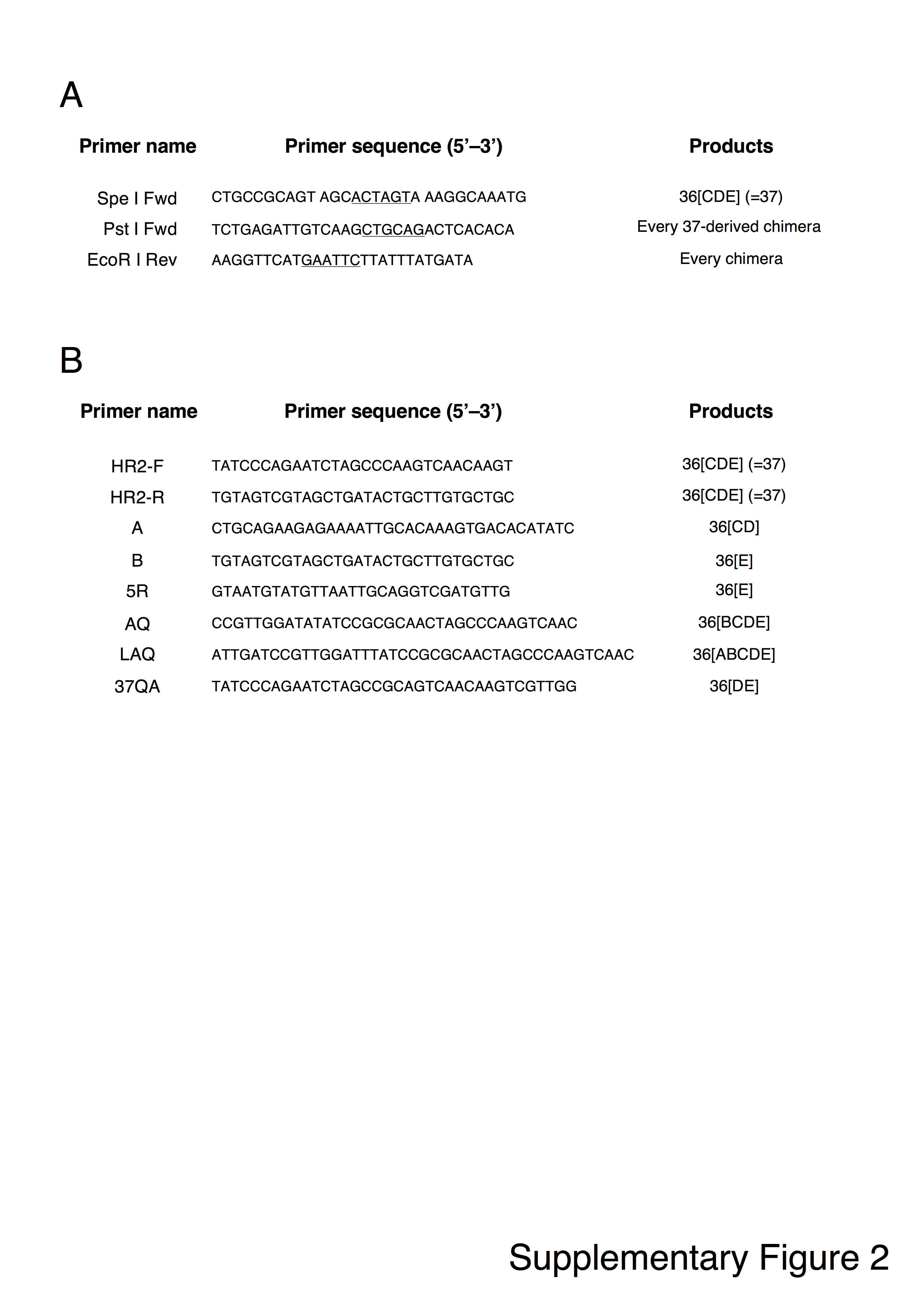

Supplement: FIGURE S2 — Nucleotide sequences of the oligonucleotide primers used for creation of chimeric F proteins. (A) Nucleotide sequences of the oligonucleotide primers which were used for inserting respective PCR fragment into the pcDL-SRa expression vector encoding the chimeric F protein, no. 36, by ligation. Restriction enzyme sites are underlined. (B) Nucleotide sequences of the oligonucleotide primers which were used for introducing chimeric junctions or mutations by PCR. For every primer, a primer with its complementary sequence was employed in order to connect the PCR fragments. [file Image_2.tif]

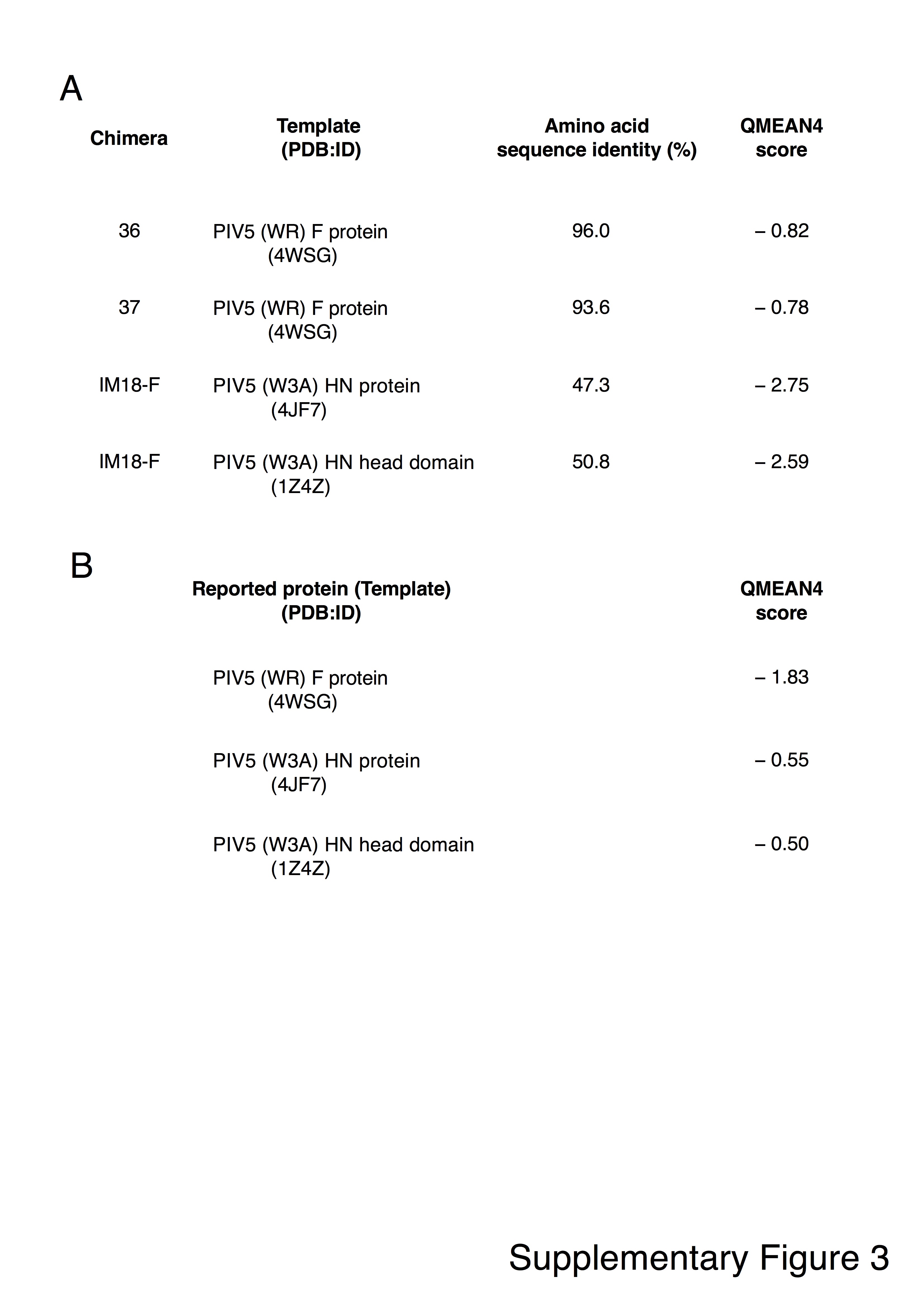

Supplement: FIGURE S3 — QMEAN scores of the model structures. QMEAN4 scores of the chimeras (A) and their templates (B) were calculated on a Web site (SWISS-MODEL). The amino acid sequence identity between each chimera and its template was also presented in (A). [file Image_3.TIF]

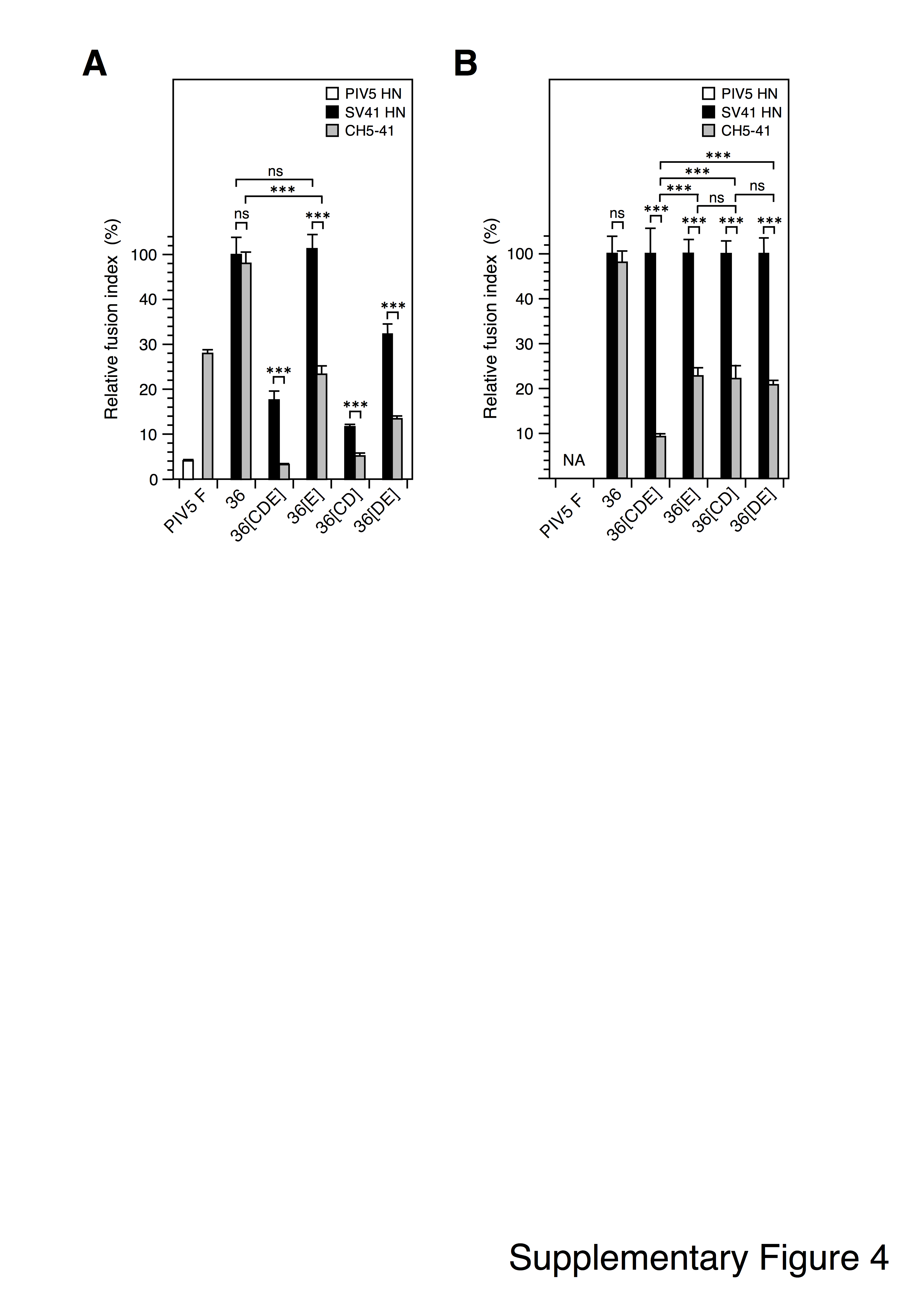

Supplement: FIGURE S4 — The F stalk domain modifies the HN protein specificity. (A) Efficiency of fusion induction by chimeric F proteins. The fusion indices given by the chimeric F proteins shown in Figure 2D are normalized to their cell surface-localization levels. The statistical significance was evaluated by one-way ANOVA as described in the section “Materials and Methods” (∗∗∗p < 0.01, n = 10). ns, not significant. (B) HN protein specificity of the F proteins. For each chimeric F protein used in Figure 2D, the fusion index given with CH5-41 was normalized to that given with the SV41 HN protein. The statistical significance was evaluated by one-way ANOVA as described in the section “Materials and Methods” (∗∗∗p < 0.01, n = 10). ns, not significant; NA, not applicable. [file Image_4.TIF]

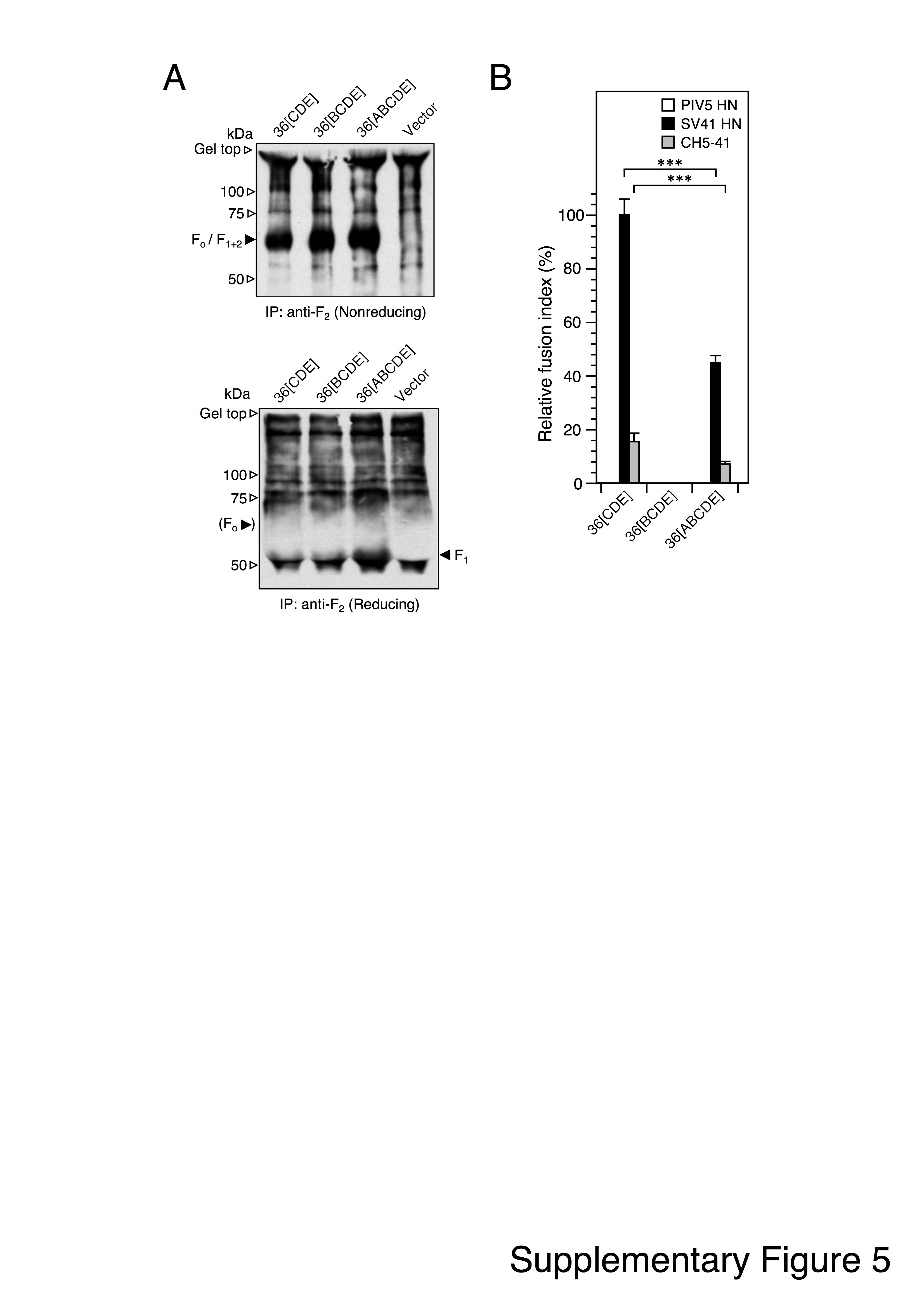

Supplement: FIGURE S5 — No. 37 is a more SV41 F-like protein than no. 36. (A) Detection of the F proteins in the plasmid-transfected cells. (Upper panel) long-exposed image of the data presented in Figure 3A; the cleaved form (F1+2) and unclevaed form (F0) are considered to comigrate with each other under non-reducing conditions. (Lower panel) under reducing conditions, the cleaved form (F1) nearly comigrates with an unidentified cellular protein (ca. 50 kDa), which have been precipitated by the ant-PIV5 F2 polyclonal antibody. In all likelihood, this 50 kDa-protein migrates much slower than F0/F1+2 under non-reducing conditions (A) due to disulfide-mediated association with other unidentified cellular protein(s). In the parenthesis is indicated the predicted position of the uncleaved form, F0. (B) Efficiency of fusion induction by the F proteins. The fusion indices given by the chimeric F proteins shown in Figure 3B are normalized to their cell surface-localization levels. The statistical significance was evaluated by one-way ANOVA as described in the section “Materials and Methods” (∗∗∗p < 0.01, n = 10). [file Image_5.tif]

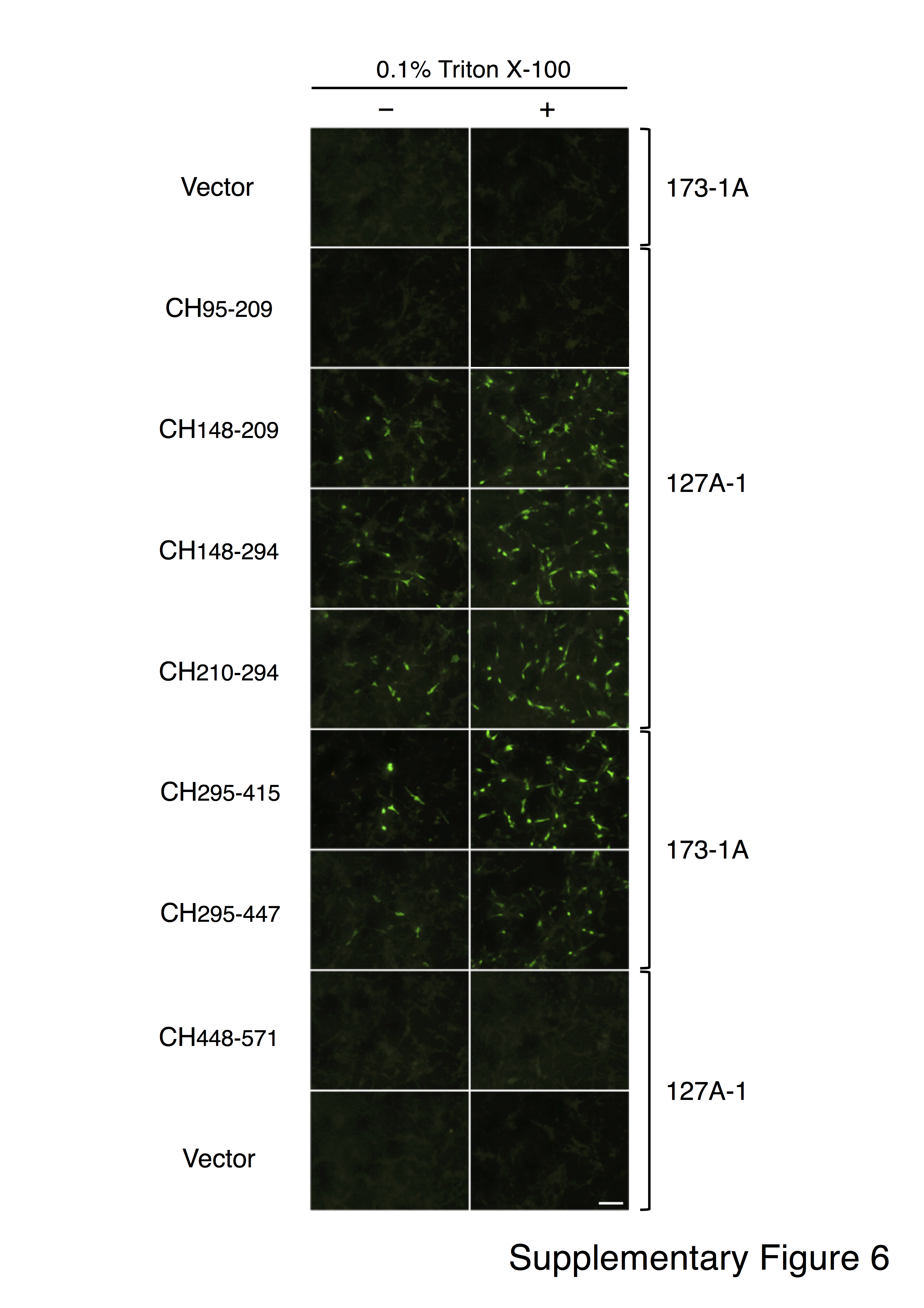

Supplement: FIGURE S6 — Immunofluorescent staining the chimeric HN proteins used in Figure 4B. Subconfluent BHK cells grown on glass coverslips in six-well culture plates were transfected with 2.0 μg/well of the pcDLSRa expression vector encoding each HN protein. After 24 h of incubation at 37°C, the cells were fixed with 4% paraformaldehyde in PBS, washed three times with PBS, and permeabilized or not permeabilized with 0.1% Triton X-100 in PBS. The HN proteins were visualized by indirect immunofluorescent staining as described in the section “Materials and Methods” by using MAb 173-1A or MAb 127A-1. Bar, 100 μm. [file Image_6.TIF]

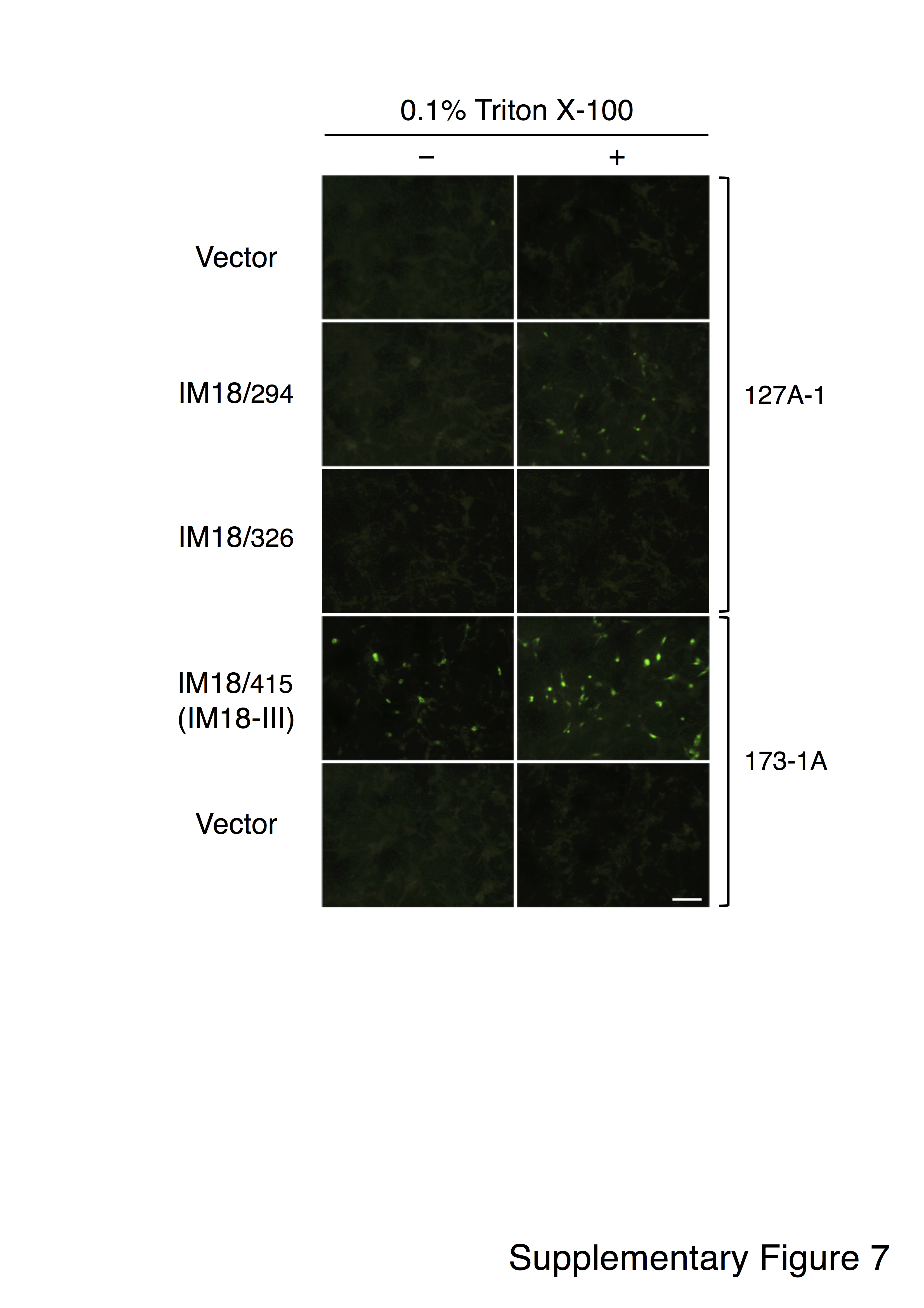

Supplement: FIGURE S7 — Immunofluorescent staining the chimeric HN proteins used in Figure 5A. Subconfluent BHK cells grown on glass coverslips in six-well culture plates were transfected with 2.0 μg/well of the pcDLSRa expression vector encoding each HN protein. After 24 h of incubation at 37°C, the cells were fixed with 4% paraformaldehyde and the HN proteins were visualized as described in the legend for Supplementary Figure S6 by using MAb 173-1A or MAb 127A-1. Bar, 100 μm. [file Image_7.TIF]

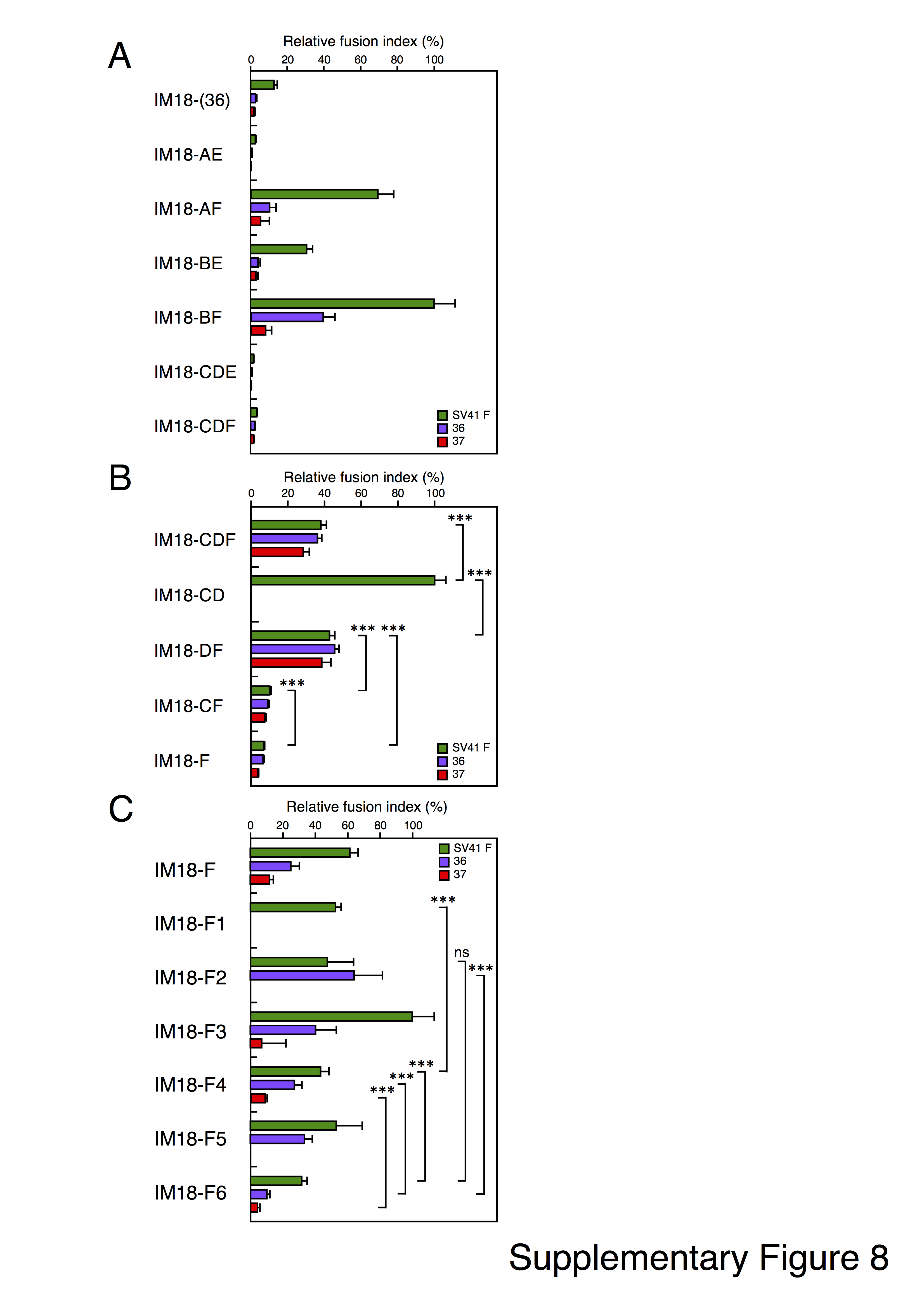

Supplement: FIGURE S8 — Efficiency of fusion promotion by the HN proteins. The fusion indices given by the HN proteins shown in Figure 6C (A), Figure 6B (B), and Figure 7C (C) are normalized to their cell surface-localization levels. The statistical significance was evaluated by one-way ANOVA as described in the section “Materials and Methods” (∗∗∗p < 0.01, n = 10). ns, not significant. [file Image_8.TIF]

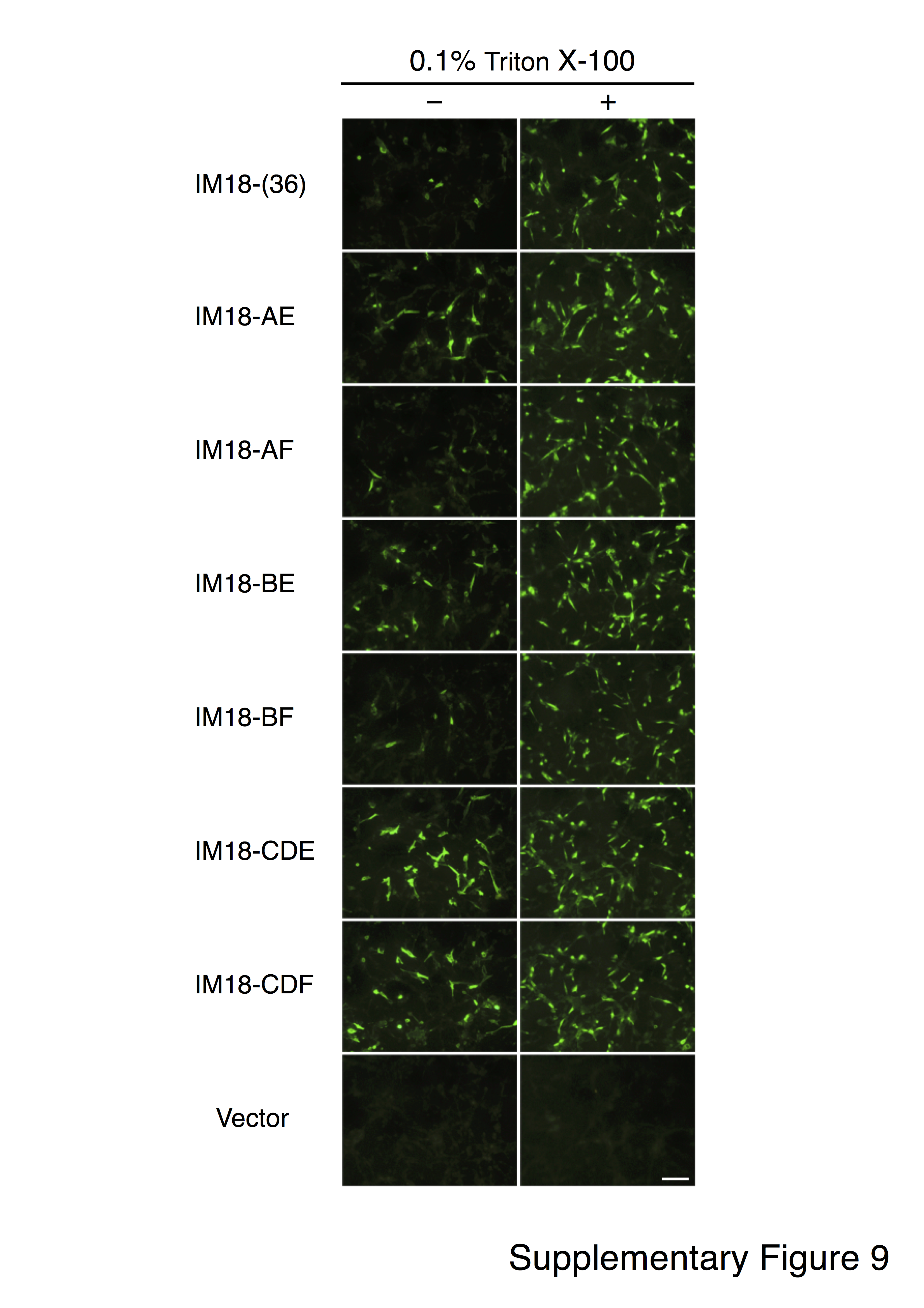

Supplement: FIGURE S9 — Immunofluorescent staining the chimeric HN proteins used in Figure 6A. Subconfluent BHK cells grown on glass coverslips in six-well culture plates were transfected with the expression vector encoding each HN protein. After 24 h of incubation at 37°C, the cells were fixed with 4% paraformaldehyde and the HN proteins were visualized as described in the legend for Supplementary Figure S6 by using MAb 173-1A. Bar, 100 μm. [file Image_9.TIF]

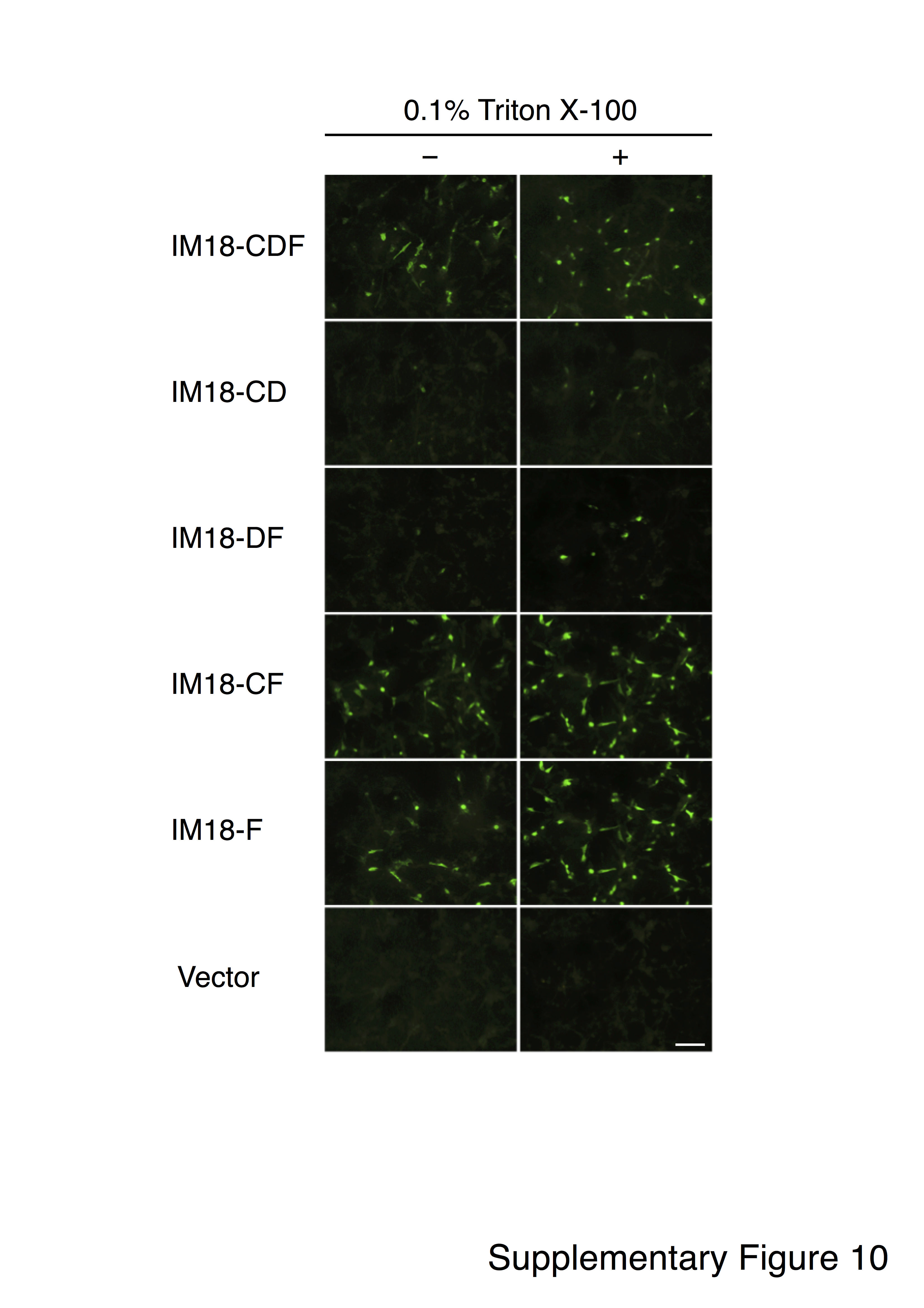

Supplement: FIGURE S10 — Immunofluorescent staining the chimeric HN proteins used in Figure 6B. Subconfluent BHK cells grown on glass coverslips in six-well culture plates were transfected with the expression vector encoding each HN protein. After 24 h of incubation at 37°C, the cells were fixed with 4% paraformaldehyde and the HN proteins were visualized as described in the legend for Supplementary Figure S6 by using MAb 173-1A. Bar, 100 μm. [file Image_10.TIF]

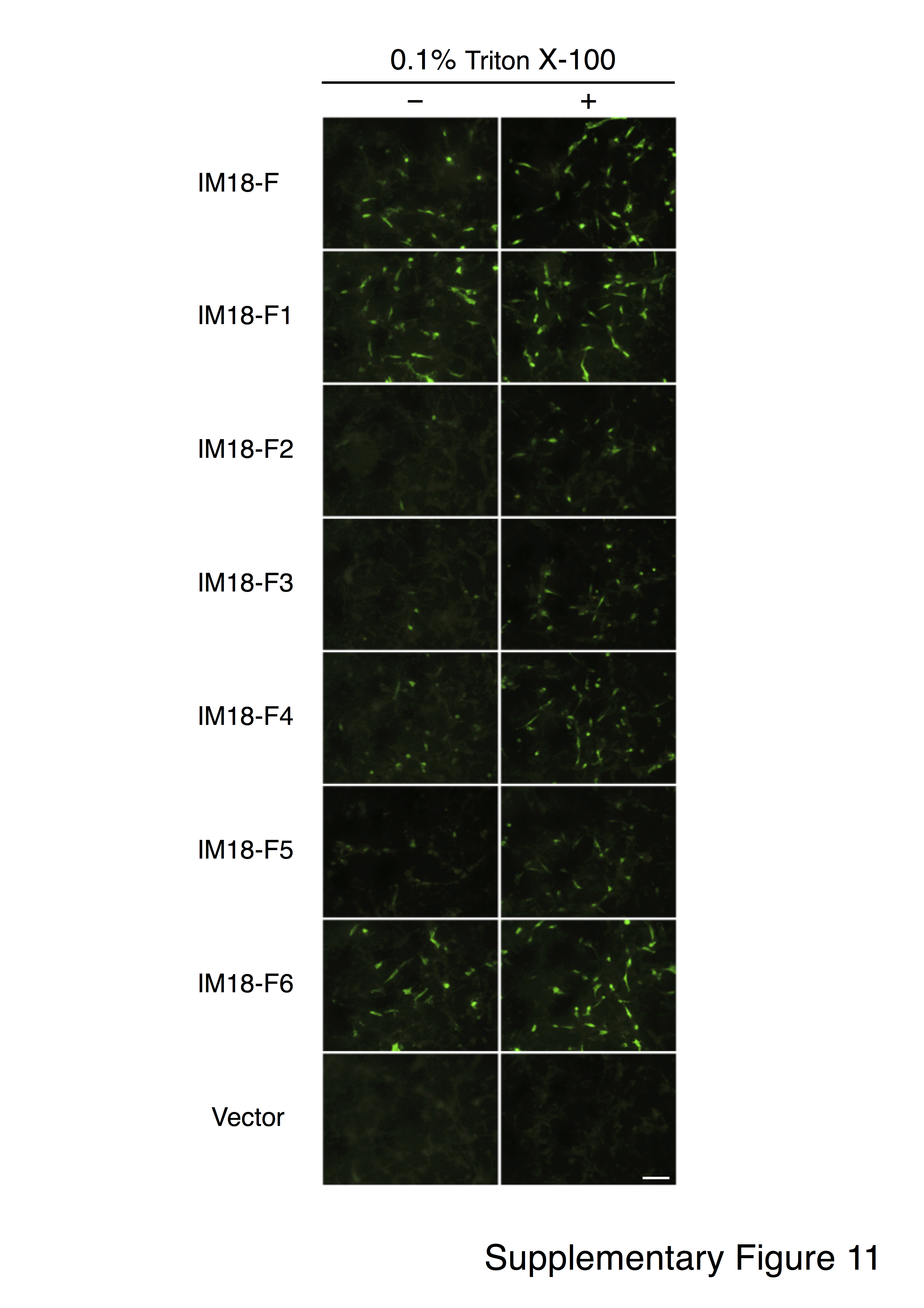

Supplement: FIGURE S11 — Immunofluorescent staining the chimeric HN proteins used in Figure 7B. Subconfluent BHK cells grown on glass coverslips in six-well culture plates were transfected with the expression vector encoding each HN protein. A After 24 h of incubation at 37°C, the cells were fixed with 4% paraformaldehyde and the HN proteins were visualized as described in the legend for Supplementary Figure S6 by using MAb 173-1A. Bar, 100 μm. [file Image_11.TIF]
